# MS brain health quality standards: a survey on the reality in clinical practice in Germany

**DOI:** 10.1186/s42466-024-00333-4

**Published:** 2024-11-18

**Authors:** Isabel Voigt, Katja Akgün, Hernan Inojosa, Judith Haas, Herbert Temmes, Sven G. Meuth, Gavin Giovannoni, Tjalf Ziemssen

**Affiliations:** 1 Center of Clinical Neuroscience, Department of Neurology, Medical Faculty and University Hospital Carl Gustav Carus, TU Dresden, Fetscherstraße 74, 01307 Dresden, Germany; 2 DMSG Bundesverband, Hannover, Germany; 3 Department of Neurology, Medical Faculty and University Hospital Düsseldorf, Heinrich-Heine-University, Düsseldorf, Germany; 4 The Faculty of Medicine and Dentistry, Blizard Institute, Queen Mary University of London, London, UK

**Keywords:** Multiple sclerosis, Disease management, Quality indicators, Survey

## Abstract

**Background:**

The quality of treatment is especially critical in the context of complex and chronic diseases such as multiple sclerosis (MS). The Brain Health Initiative, an independent international consortium of neurologists, reached a consensus on time-based quality standards prioritizing brain health-focused care for people with MS.

**Objectives:**

To gain deeper insights into the transferability of these quality standards to a specific area, we conducted a survey among MS experts across various MS centers in Germany.

**Methods:**

Participants were asked about time frames considered high standards and those currently being implemented in daily routine based on their experience.

**Results:**

The results reveal a large gap between ideal conceptions and their adaptation in the real world, mostly due to a lack of resources.

**Conclusions:**

Nevertheless, these guidelines and recommendations can be aspired to as ideals. Consensual and inclusive clinical pathways complemented by measurable quality indicators are needed to improve care and approach these ideals. Neither exists in the current management of MS.

**Supplementary Information:**

The online version contains supplementary material available at 10.1186/s42466-024-00333-4.

## Introduction

Quality in the provision of healthcare services is an overarching goal that transcends borders and cultures. It is especially critical in the context of complex and chronic diseases such as multiple sclerosis (MS). As a highly complex chronic disease, MS may require lifelong treatments with constant monitoring and adjustments. The pursuit of ideal care and treatment for people with MS (pwMS) has led to the development of international recommendations, local guidelines and consensus standards aimed at improving treatment and quality of life for pwMS [1–5]. There is widespread agreement on the importance of early diagnosis and treatment, which leads to an improvement in the physical symptoms reported by patients and to a reduction in long-term disability and costs of illness [6–8], although there are some barriers to early treatment [9, 10]. Due to the complex and multidimensional nature of MS, pwMS should be treated by different specialists, e.g., within the framework of an MS care unit [4], an interdisciplinary treatment group applying standardized diagnostic and therapeutic algorithms to pwMS.

Another significant contribution to ideal care is the joint work of the Brain Health Initiative (BHI, www.msbrainhealth.org), an independent international consortium of neurologists, to forge a consensus on quality standards prioritizing brain health-focused care for pwMS [1]. They designed three main recommendations outlining strategies for MS diagnosis and therapy. First, delays in diagnosis and treatment initiation or optimization should be avoided. Second, disease activity should be monitored in detail and closely to implement a treat-to-target therapeutic approach [11]. Third, robust scientific evidence will be generated from real-world data, which in turn can be used to optimize personalized MS therapy [12]. From these broad quality recommendations, a working group within the BHI has developed specific time-based quality standards for a brain-healthy MS treatment across the care pathway. The quality standards were divided into five phases of the care pathway: *referral and diagnosis*, *priorities following diagnosis*, *routine monitoring and support*, *treatment decisions*, and *new symptoms*. These phases were accompanied by timeframes, which were defined as *core*, *achievable* and *aspirational standard levels.* The *core standard of care* represents the minimum level of care worldwide, regardless of the local health care system (minimum standard), the *achievable standard of care* is a realistic goal for most MS providers (good standard), and the *aspirational standard of care* is achieved by providers with sophisticated health care systems (high standard) [13].

However, as healthcare systems and patient needs differ from country to country, the BHI has developed a quality improvement tool and tested it in various MS centers around the world, including the Center for Clinical Neuroscience (ZKN) Dresden, Germany [14]. The aim of our paper was to gain even deeper insights into the transferability of the international benchmarks of the BHI to the real healthcare situation in Germany. For this purpose, we conducted a survey on the perspectives of MS experts in different German MS centers and summarized their experiences and expectations. Our research seeks to bridge the gap between international standards and local practice, ultimately aiming to improve the quality of life of pwMS in Germany.

## Materials and methods

**Fig. 1:**
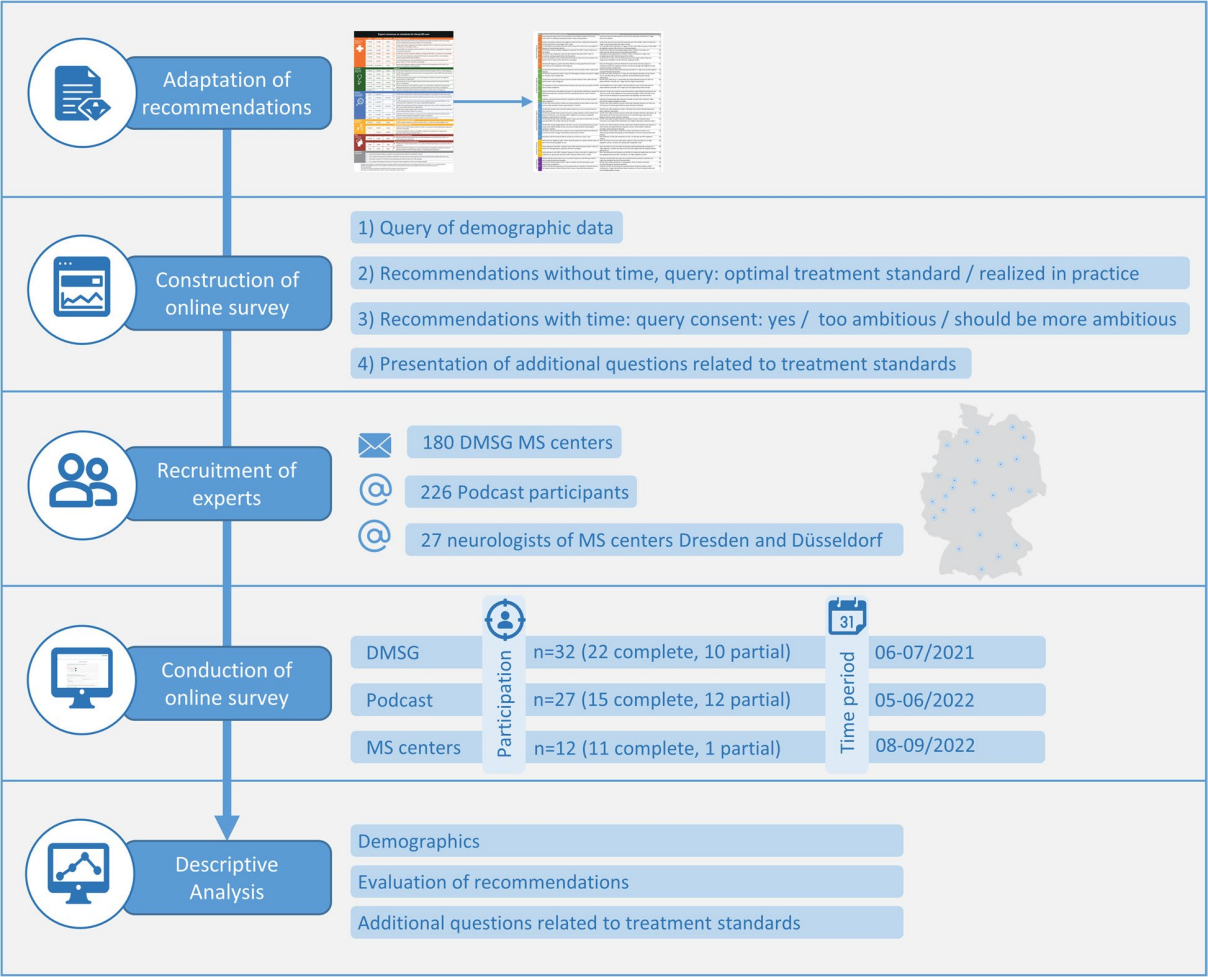
Methodological approach

We conducted an online survey in which we pursued the issues of what time frames neurologists in Germany consider to be a high standard, which time frames are currently used in their daily practice and how they rate the *aspirational standard of care* of BHI (Fig. 1).

### Adaptation of recommendations

We first adapted the quality standards to the German language (Supplement 1) and put them into a clear and understandable form to be presented within the five phases of the care pathway. Additionally, other questions were integrated into the corresponding phases, namely, whether someone who has symptoms of MS for the first time should present to a primary care physician or to a neurologist; whether an initial MRI should be performed before the initial presentation to a neurologist; and whether the surveyed neurologist performs MRI, cognitive screening, and brain-healthy lifestyle counseling on his or her patients. The online survey was conducted with the software *lime survey* and consisted of a query of demographic data and statements with associated time frames to be evaluated.

### Evaluation criteria

The BHI statements were presented in order, step by step. First, a statement without the BHI-defined time frame was presented, and neurologists were asked what time frame they considered a high standard for this statement. Similarly, they were asked to indicate what time frames were currently being implemented in their daily routine based on their experience. Next, neurologists were shown the *aspirational standard of care* defined by the BHI and asked to rate it: do they agree, do they think it is too ambitious, or should it be more ambitious? In addition, the experts were able to comment on each statement with the associated time frame in the free text section.

### Conduction of the survey

Experts were selected based on their research, implementation of projects and professional experience in MS. The survey was conducted with three different groups of experts to represent the greatest possible diversity. First, in cooperation with the German Multiple Sclerosis Society (DMSG), representatives of the 180 DMSG-recognized MS centers were invited by letter to participate in the survey. They received information about the aim of the study, instructions to rate the statements and a QR code for participation. The survey was hosted by the DMSG between June and July 2021. The data were subsequently transferred to the research team for analysis.

In a second round, neurologists who participated in the podcast series broadcast by the ZKN Dresden (https://www.youtube.com/c/zkndd) were invited, and in a third round, neurologists working at the ZKN Dresden and at the neurological university clinic Düsseldorf were invited. They received a personal invitation via e-mail and received information about the aim of the study, instructions to rate the statements with associated time frames, and a personalized link to conduct the survey. Reminders were sent if necessary. Both the second and third rounds of the survey were conducted by ZKN Dresden between May and September 2022.

### Statistical analysis

The data were anonymized for analysis. Only datasets with complete demographic data and complete responses to the first question were selected. Dropouts were identified and successively excluded from the analysis, with the corresponding sample size (*n)* indicated for each question. Descriptive statistics were applied to the dataset, e.g., for analyzing demographic variables and survey items, including the mean, median or range, as appropriate. The free-text comments were abstracted, and their content was selectively reproduced for analysis.

## Results

In sum, 71 of the 433 German experts who were approached participated in our study, including 32 representatives invited via DMSG, 27 podcast participants and 12 neurologists from the MS centers in Dresden and Düsseldorf (Fig. 1). On average, the experts were 49.1 (± 10.4) years old and had 21 (± 10.3) years of professional experience, thereof 13.9 (± 9.2) years specialized in MS. Approximately 60% had practiced in a large city, while the others had practiced in small towns and rural areas. Almost two-thirds (65%) practiced in a clinic; one-third (33%) practiced in a private practice, a medical care center, or a rehabilitation center; and one neurologist was retired (Table 1).

**Table 1:** Characteristics of the panel of experts

	*n* = 71
**Age** (years)	
Mean (SD)	49.1 (10.4)
Median (range)	49 (29–73)
**Professional practice duration** (years)	
Mean (SD)	21.1 (10.3)
Median (range)	13 (2–47)
**MS specialized practice duration** (years)	
Mean (SD)	13.9 (9.2)
Median (range)	20 (0–35)
**Practice setting**, n (%)	
Urban/major city (> 100.000 residents)	42 (59.2%)
Rural/small city (< = 100.000 residents)	29 (40.8%)
**Practice organization**, n (%)	
Licensed	22 (31.0%)
Self-employed	17 (23.9%)
Medical care center	5 (7.0%)
Clinic	46 (64.8%)
Outpatient	25 (35.2%)
Inpatient	20 (28.2%)
Out- and inpatient	1 (1.4%)
Retirement	1 (1.4%)
Rehabilitation clinic	2 (2.8%)
**Participation in MS registry**, n (%)	
Yes	39 (54.9%)
No	20 (28.2%)
N/S	12 (16.9%)
**DMSG certification**	
MS-center	28 (39.4%)
MS-focused center	10 (14.1%)
MS-rehabilitation center	2 (2.8%)
No DMSG certification	19 (26.8%)
N/S	12 (16.8%)
**Participation in studies**, n (%)	
Yes	42 (59.2%)
No	17 (23.9%)
N/S	12 (16.9%)

### Referral and diagnosis

**Fig. 2:**
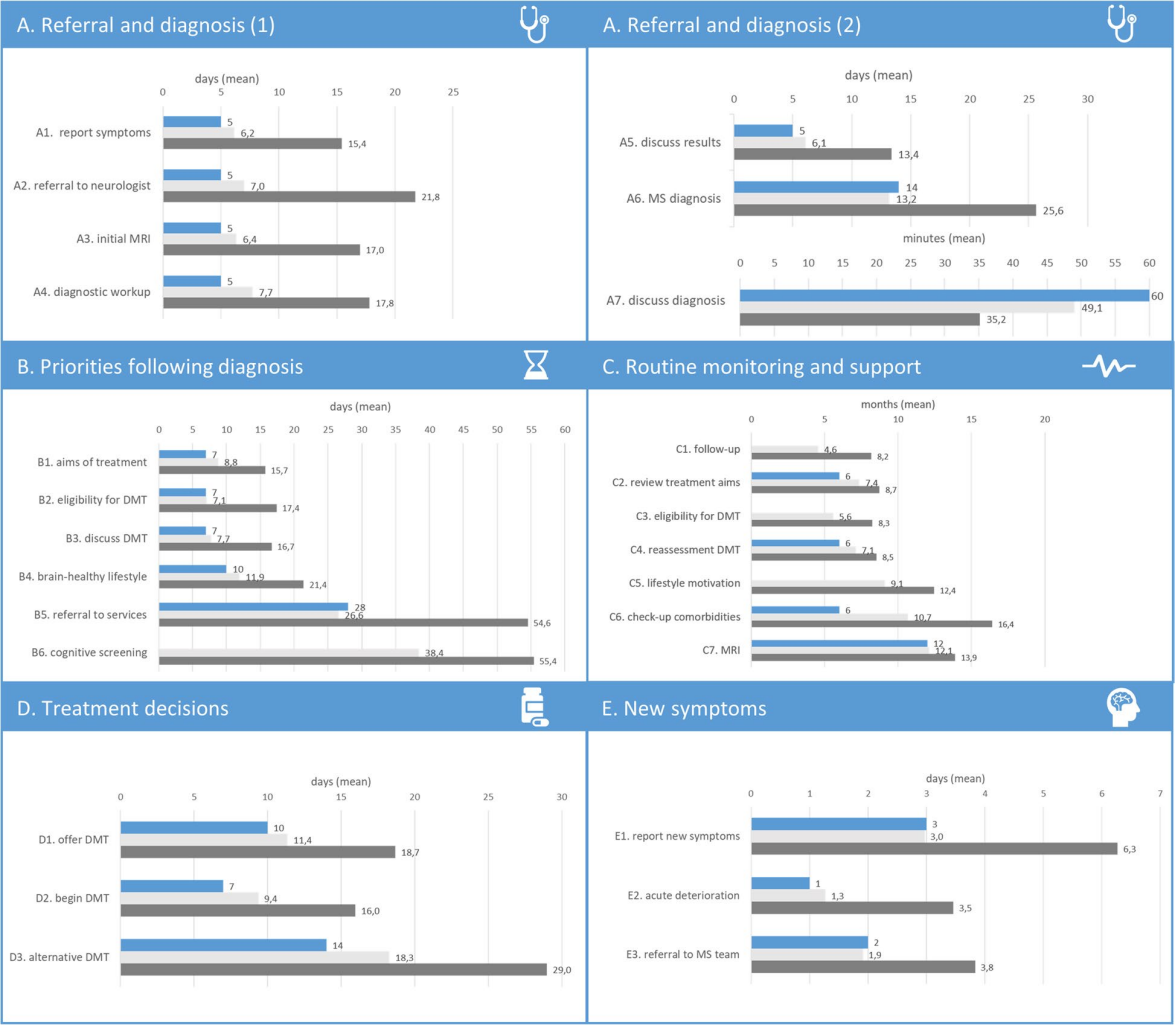
Results of evolution (BHIs aspirational standard, expert opinion: high standard, expert experience: currently realized in daily routine). Caption Fig. 2: blue = BHIs aspirational standard, light grey = expert opinion: high standard, dark grey = expert experience: currently realized in daily routine

For all items, expert opinions on high standard periods differ from BHI time frames for an *aspirational standard of care*. Experts indicate an average of 1.3 days more than the BHI does. However, a much more noteworthy deviation is seen when comparing time frames currently realized in daily routine with BHI standards. On average, it takes 12 more days in daily routine than the BHI standard specifies (Fig. 2).

**Fig. 3:**
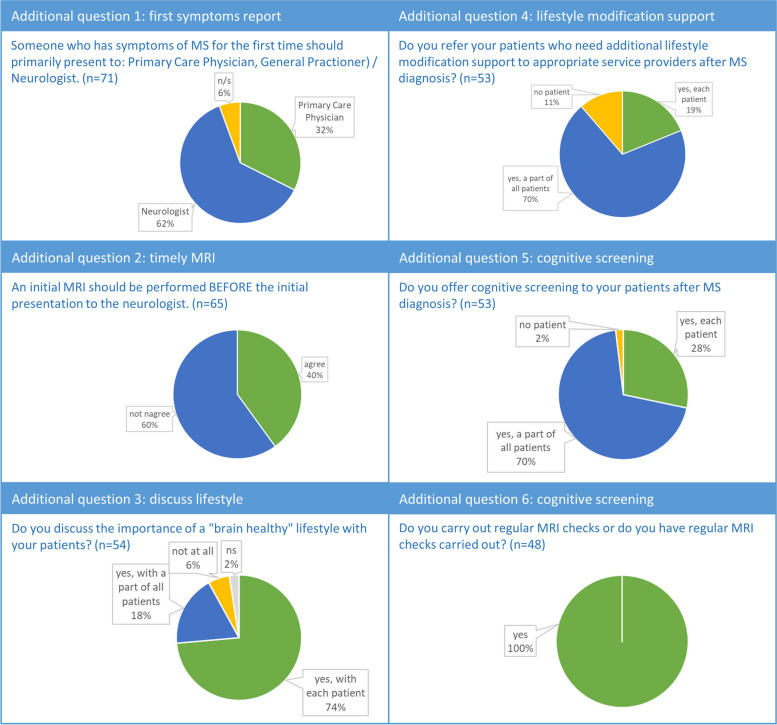
Results of additional questions

Nevertheless, approximately three-quarters (73–81%) of the experts agreed with the *aspirational standard of care* of the BHI, with the exception of statement A7 on how long an initial consultation to discuss the diagnosis with the patient should be. Many neurologists found 60 min too ambitious because it is not realizable in daily routine and would possibly overtax the patient. Instead, two appointments should be made (Supplement 2). For the additional question, "Someone who has symptoms of MS for the first time should present primarily to a primary care physician or a neurologist" nearly two-thirds (62%) were in favor of the patient presenting primarily to a neurologist; just over one-third (32%) voted for the primary care physician, and 6% did not indicate this (Fig. 3). Three-fifths agreed with the additional statement "An initial MRI should be performed before the initial presentation to the neurologist", and two-fifths disagreed (Fig. 3).

### Priorities following diagnosis

Like in the previous topic block, expert opinions on high standard periods differ from BHI time frames for an *aspirational standard of care*; experts indicate an average of 0.6 days more than the BHI does. Statement B5 (additional support for lifestyle modifications) is the only one for which the experts define 1.4 days less time as a high standard than the BHI. For item B6 (cognitive screening), the BHI did not provide an *aspirational standard of care*. The time frames that are currently realized in daily routine practice deviate markedly from the BHI standard for an *aspirational standard of care*. On average, it takes 13.4 days more in daily routine than the BHI standard specifies (Fig. 2).

Most experts (64–73%) agreed with the *aspirational standard of care* of the BHI, with the exception of statement B4 (time frame for discussion of a “brain-healthy” lifestyle). Only half of the neurologists (54%) agreed, one quarter of the participants found 10 days too ambitious, partly because they did not consider the issue of a “brain-healthy” lifestyle to be important or would prefer to subordinate it to what they considered to be more important issues such as DMT (Supplement 2).

For the additional question, "Do you discuss the importance of a ‘brain-healthy’ lifestyle with your patients?" nearly three-quarters (74%) answered “yes, with every patient”, just under one fifth (18%) answered “yes, with a part of all patients”, 6% did not discuss that at all, and 2% did not indicate. 70% of the neurologists referred a portion of their patients who needed additional lifestyle modification support to appropriate service providers after MS diagnosis; a good fifth (19%) referred to all of their patients; and 11% did not refer any of their patients, among other things, because no sufficient infrastructure existed. Additionally, 70% of the participants offered cognitive screening to a subset of their patients, 28% offered this to all of their patients, and 2% did not offer this at all. Some neurologists expressed the opinion that cognitive screening should be performed only when symptoms are present, while others criticized that it is not always feasible due to a lack of resources (Fig. 3).

### Routine monitoring and support

For the timeframes in the monitoring process, experts defined the high standard on average as 2.4 months more than the BHI does. In daily routine, it takes on average 5.2 months longer than the BHI defines as an *aspirational standard of care* (Fig. 2). For three items (C1. follow-up, C3. eligibility for DMT, and C5. lifestyle motivation), the BHI did not provide an *aspirational standard* but rather an *achievable standard of care*. In these cases, we did not ask for agreement because our focus was on experts’ opinions on *aspirational standards of care*. Agreement on BHI’s items C2. review treatment aims (69%), C4. reassessment DMT (67%), and C6. check-up comorbidities (59%) was fairly high; approximately one-fifth thought that the time frames of BHI were too ambitious. Eighty-eight percent of the neurologists agreed that all MS patients should be offered an MRI scan at least once every 12 months (C7), and only 5% thought that this was too ambitious (Supplement 2).

According to the free-text comments, it was clear that some of the neurologists would prefer to specify the time periods for reviewing treatment goals (C2), currently prescribed DMT (C4), or reviewing comorbidities (C6) based on certain factors, e.g., disease activity and progression, rather than defining fixed time periods for those (Supplement 2). The additional question of whether the respondent performs or performed regular MRI was answered “yes” by all without exception (Fig. 3).

### Treatment decisions

For the timeframes concerning treatment decisions, experts defined the high standard on average as 2.7 days more than the BHI does. In daily routine, it takes on average 10.9 days longer than the BHI defines as an *aspirational standard of care* (Fig. 2). Agreement on all the BHI items was quite high (67–77%). For items D1. offer DMT and D3. alternative DMT, the neurologists who disagreed, either thought the time periods were too ambitious or not ambitious enough. The neurologists, who disagreed with item D2. begin DMT, thought the time period was too ambitious, first because it was difficult to make an appointment within 14 days and second because various preliminary examinations had to occur, for which the time period was rather too short (Supplement 2).

### Evaluation of “New symptoms” items

For timeframes dealing with new symptoms, experts defined the high standard on average as 0.1 days more than the BHI does. In daily routine, it takes on average 2.5 days longer than the BHI defines as an *aspirational standard of care.* Agreement on all the BHI items was quite high. For items E1. report new symptoms and E3. referral to MS team, the neurologists who disagreed tended to think that the time frames were not ambitious enough. For item E2. acute deterioration, the neurologist who disagreed, thought that the time period was too ambitious without providing a reason (Supplement 2).

## Discussion

In this paper, we present the results of a survey among neurologists in Germany on the transferability of the quality standards for timely MS care established by the BHI. The BHI itself has already tested its quality improvement tool with those involved in the development of consensus standards in pilot studies in different countries [14]. However, little research has been conducted on how other neurologists with MS expertise think about quality standards and their applicability in practice. Our intention was not to question the quality standards but rather to examine the extent to which they can be integrated into the real healthcare situation in Germany and what obstacles there are in doing so. Therefore, we have also integrated additional questions that ask, e.g., about the current implementation of certain assessments.

In our cohort, 71 participating neurologists across Germany had an average age of 49 years and had specialized in MS care for approximately 14 years, mostly in an outpatient or clinical setting. The time periods indicated by experts as high standards were broadly similar to those *aspirational standards of care* envisioned by BHI. It is all the more remarkable that the timeframes currently realized in the daily routine of the surveyed experts differ meaningfully from those *aspirational standards of care* of the BHI. There were considerable differences in most cases, revealing that there is a large gap between ideal conceptions and their adaptation in the real world. The time frames currently realized in daily routine were mostly twice as long as the *aspirational standards of care* proposed by the BHI. This finding coincides with the results of other studies, which also show a gap between guidelines or recommendations and implementation in the reality of care [15, 16]. Free-text comments indicate that some neurologists believe that most time slots are not feasible in daily routine care due to lack of resources, even if they agree with the aspirational time frames suggested by the BHI. Some neurologists noted that patients may be overwhelmed with certain information. Others feel that teaching a “brain-healthy” lifestyle should play more of a secondary role because other topics are more important, e.g., starting a DMT. Referral to other service providers or cognitive screenings is not feasible from the perspective of many neurologists due to nonexistent or insufficient resources and infrastructure.

This survey has a few limitations to mention. The number of respondents was relatively small. This is due to the fact that there are 9,636 outpatient and inpatient neurologists in Germany who treat more than 20 conditions in the most cases, including dementia, dystonia, dizziness, migraine, Parkinson's disease and stroke [17]. Accordingly, only a small portion of them specialize in multiple sclerosis (MS). Our aim was to contact as many of these neurologists specialized in MS, who regularly treat a large number of pwMS, to participate in our survey. To achieve this, we used the networks of the German MS Society (DMSG) and established academic contacts in both the outpatient and inpatient sectors to obtain a comprehensive perspective. Not all of the contacted neurologists participated in the survey, which is a common challenge when conducting surveys, particularly among physicians who may have limited time to respond [18–20]. Although our survey is not statistically representative, key parameters within our participant panel closely resemble the distribution seen across Germany. For example, two thirds of neurologists in our survey work in the inpatient sector, while one third work in the outpatient sector, mirroring the national distribution. Similarly, the age distribution of our participants aligns with that of the broader neurology community in Germany [17]. We also successfully engaged neurologists from 10 out of 16 federal states in Germany to participate in the survey, ensuring diverse geographic representation despite the non-random sampling. Twenty-three of the participants gradually dropped out during the survey (71 participants in the first question vs. 48 participants in the last question). We have therefore also listed the number of participants for each question (Supplement 1). The survey was administered in three stages at different time points, which may have caused further bias. Those aspects led us to perform purely descriptive analyses.

Despite a relatively low response rate, insights from those who responded are incredibly valuable. Even with a small sample size, they may uncover important trends, challenges or innovations in the field of MS neurology. The usability of these results is beyond question, as it becomes clear that there are large differences between the time frames that are considered ideal and the time frames that can be realized in everyday routine care.

## Conclusion

There are two take-home messages in our survey. First, it seems difficult to propose guidelines and recommendations that are valid in an international context, and second, it is just as difficult to put them into daily routine care. Nevertheless, these guidelines and recommendations can be aspired to as ideals. Likewise, improving and expanding the necessary resources and infrastructure can be a step in the right direction. In Germany, the *Gemeinsame Bundesausschuss (G-BA)* introduced the so-called *Ambulante Spezialfachärztliche Versorgung (ASV)* at the end of 2022 [21, 22]. Its framework conditions correspond only to the concept of the minimum requirements of Soelberg Sorensens MS Care Unit [4, 23].

Against the background of our survey, the question arises as to what extent and how such MS care units could be implemented in Germany. Most likely, only a few centers, especially academic MS clinics that already care for pwMS in outpatient settings, will succeed in doing so, and it is hoped that in the course of digitalization and the use of telemonitoring, patients in rural areas can be adequately cared for. To improve care and approach aspirational goals, as mentioned by the BHI, consensual and inclusive clinical pathways are needed that are regularly adapted to the current state of research and describe the phases of care along the intersectoral and interorganizational pathways of patients in a network of service providers. These clinical pathways, supplemented by quality indicators, can ensure the best possible care for pwMS [24]. At present, however, there are neither consensual clinical pathways nor meaningful and measurable quality indicators for MS care that could set new standards for MS care when implemented in practice [25]. With the ASV-MS, the German health system has taken the path of opening up to the medical practice (licensed) sector, following the directive on outpatient treatment in hospitals (*Richtlinie über die ambulante Behandlung im Krankenhaus, ABK-RL*). However, the high complexity of MS therapy will continue to raise the question of resources and thus the issue of further specialization.

## Supplementary Information

Supplementary Material 1.

Supplementary Material 2.

## Data Availability

The datasets generated during and/or analyzed during the current study are available from the corresponding author upon reasonable request.

## Abbreviations

ABK-RL: Richtlinie über die ambulante Behandlung im Krankenhaus (directive on outpatient treatment in hospital)
ASV: Ambulante Spezialfachärztliche Versorgung (Outpatient specialist care)
BHI: Brain Health Initiative
DMSG: German Multiple Sclerosis Society
ERDF: European Regional Development Fund
GBA: Gemeinsamer Bundesausschuss (Joint Federal Committee)
MS: Multiple sclerosis
pwMS: People with multiple sclerosis
QPATH4MS: Quality Management in MS Care by Clinical Pathways
SD: Standard deviation
ZKN: Center for Clinical Neuroscience
