# Supplementary material for: MS brain health quality standards: a survey on the reality in clinical practice in Germany
Source: Neurol Res Pract. 2024 Nov 18;6:59. doi: 10.1186/s42466-024-00333-4 (PMC11571952; doi:10.1186/s42466-024-00333-4)
Supplement: Supplementary file 1 — Supplementary Material 1. [file 42466_2024_333_MOESM1_ESM.pdf]

	#	Original [high standard]; Hobart (2018)	Brain Health survey 2021/2022	short form	n
referral and diagnosis	A1	Anyone experiencing for the first time symptoms that might be related to MS should report them to a healthcare professional within 5 days of noticing them.	Jemand, der das erste Mal Symptome einer MS hat, sollte diese innerhalb von 5 Tagen einem Arzt mitteilen.	A1. report symptoms	71
	A2	Anyone who reports symptoms that might be related to MS to a healthcare professional should be referred to a neurologist within 5 days.	Jemand, der einem Arzt das erste Mal Symptome einer MS schildert, sollte innerhalb von 5 Tagen zu einem Neurologen überwiesen werden.	A2. referral to neurologist	71
	A3	An initial MRI scan should be performed within 5 days of first referral to a neurologist for diagnosis (if not performed earlier).	Ein erstes MRT sollte innerhalb von 5 Tagen nach der ersten Überweisung zum Neurologen durchgeführt werden (falls nicht schon früher geschehen).	A3. initial MRI	67
	A4	The MS team should complete a diagnostic workup for MS within 7 days of referral to a neurologist.	Das MS Team sollte die notwendigen diagnostischen Verfahren innerhalb von 7 Tagen nach der Überweisung zum Neurologen abschließen.	A4. diagnostic workup	66
	A5	The results from a diagnostic workup for MS should be discussed within 5 days of completion, during an appointment with the patient.	Die Ergebnisse der diagnostischen Verfahren sollten innerhalb von 5 Tagen nach Fertigstellung mit dem Patienten besprochen werden.	A5. discuss results	66
	A6	An accurate diagnosis of (uncomplicated) MS should be made and communicated to the patient within 2 weeks of their referral to a neurologist.	Innerhalb von 14 Tagen nach der Überweisung zum Neurologen sollte eine genaue Diagnose der MS gestellt und dem Patienten mitgeteilt werden.	A6. MS diagnosis	65
	A7	Following MS diagnosis, patients should be offered an initial appointment of at least 1 hour to discuss the implications of the diagnosis.	Nach der MS Diagnose sollte den Patienten ein erster Termin mit einer Länge von mindestens 60 Minuten angeboten werden, um die Auswirkungen der Diagnose und das weitere Procedere zu besprechen.	A7. discuss diagnosis	65
priorities after diagnosis	B1	The MS team should discuss the aims of treatment with each patient within 7 days of MS diagnosis.	Das MS Team sollte die Ziele der Behandlung innerhalb von 7 Tagen nach der MS Diagnose mit jedem Patienten besprechen.	B1. aims of treatment	59
	B2	The MS team should assess within 7 days of an MS diagnosis whether the patient is eligible for treatment with a suitable DMT.	Das MS Team sollte innerhalb von 7 Tagen nach der Diagnose beurteilen, ob der Patient sich für eine Behandlung mit einer passenden verlaufsmodifizierenden Therapie (DMT) eignet.	B2. eligibility for DMT	59
	B3	The MS team should discuss the pros and cons of early treatment with a DMT with each patient within 7 days of diagnosis.	Das MS Team sollte die Vor- und Nachteile der frühen Behandlung mit einer DMT mit jedem Patienten innerhalb von 7 Tagen nach der Diagnose besprechen.	B3. discuss DMT	58
	B4	The importance of a brain-healthy lifestyle should be discussed with each patient with MS within 10 days of diagnosis.	Die Wichtigkeit eines "brain healthy" Lebensstils (Nikotin, Sport, Ernährung) sollte mit jedem Patienten innerhalb von 10 Tagen nach der Diagnose besprochen werden.	B4. brain-healthy lifestyle	57
	B5	Patients with MS who need additional support to make lifestyle modifications, beyond that offered by the MS team, should be referred to appropriate services within 4 weeks of diagnosis.	Patienten mit MS, die zusätzliche Unterstützung bei der Lebensstiländerung brauchen, die über die vom MS Team angebotene Unterstützung hinausgeht, sollten innerhalb von 28 Tagen nach der MS Diagnose an entsprechende Leistungsträger vermittelt werden.	B5. referral to services	56
	B6	Cognitive screening should be offered to all patients with MS within [*no high standard*] days of diagnosis.	Kognitives Screening sollte allen Patienten innerhalb von [*kein hoher Standard*] Tagen nach der MS Diagnose angeboten werden.	B6. cognitive screening	53
routine monitoring and support	C1	The MS team should perform a follow-up clinical evaluation of each patient at least once every [*no high standard*] months. (good standard: 6 months)	Das MS Team sollte mindestens einmal in [*kein hoher Standard*] Monaten ein Follow-up der klinischen Einschätzung durchführen. (guter Standard: 6 Monate)	C1. follow-up	52
	C2	The MS team should review with each patient at least once every 6 months the aims of their treatment.	Das MS Team sollte mindestens einmal in 6 Monaten die Ziele der MS-Behandlung mit jedem Patienten überdenken	C2. review treatment aims	52
	C3	The MS team should review at least once every [*no high standard*] months whether each patient with MS who is not receiving a DMT is eligible for one, based on applicable guidelines. (good standard: 6 months)	Das MS Team sollte mindestens einmal in [*kein hoher Standard*] Monaten überdenken, ob jeder Patient mit MS, der keine DMT erhält, sich für eine DMT eignet, basierend auf gültigen Leitlinien. (guter Standard: 6 Monate)	C3. eligibility for DMT	50
	C4	The MS team should review with each patient at least once every 6 months their currently prescribed DMT and consider alternatives if possible.	Das MS Team sollte mit jedem Patienten mindestens einmal in 6 Monaten deren momentan verschriebene DMT überdenken und mögliche Alternative in Betracht ziehen.	C4. reassessment DMT	52
	C5	The MS team should engage patients with MS in an active, documented discussion about living a brain-healthy lifestyle at least once every [*no high standard*] months.(good standard: 6 months)	Das MS Team sollte mindestens einmal in [*kein hoher Standard*] Monaten die Patienten in einem aktiven und dokumentierten Gespräch zu einem "brain healthy" Lebensstil ermutigen. (guter Standard: 6 Monate)	C5. lifestyle motivation	51
	C6	All patients with MS should have a check-up with an appropriate healthcare professional to screen for and/or manage comorbidities at least every 6 months.	Alle Patienten mit MS sollten mindestens einmal in 6 Monaten ein Check-Up zur Überprüfung und/oder Behandlung von Komorbiditäten von einem entsprechenden Arzt erhalten.	C6. check-up comorbidities	51
	C7	All patients with MS should be offered an MRI scan at least once every 1 year.	Allen Patienten mit MS sollte mindestens einmal in 12 Monaten ein MRT angeboten werden.	C7. offer MRI	50
treatment decisions	D1	Patient becomes eligible for DMT: A DMT should be offered to a patient with MS within 10 days of their becoming eligible for one.	Wenn der Patient sich für eine DMT eignet, sollte ihm diese innerhalb von 10 Tagen angeboten werden, nachdem der E.ignung dafür festgestellt wurde	D1. offer DMT	48
	D2	Patient decides to start DMT: Treatment with a DMT should commence within 7 days of a patient with MS agreeing this approach with their neurologist.	Wenn der Patient sich für eine DMT entscheidet, sollte die Behandlung innerhalb von 7 Tagen beginnen, nachdem der Patient mit seinem Neurologen über das Vorgehen überein gekommen ist.	D2. begin DMT	48
	D3	Patient decides to start DMT: If patient's response to their current DMT is judged to be suboptimal, an appropriate alternative DMT should be offered within 2 weeks.	Wenn das Ansprechen des Patienten auf die DMT als suboptimal eingeschätzt wird, sollte eine geeignete alternative DMT innerhalb von 14 Tagen angeboten werden.	D3. alternative DMT	48
new symptoms	E1	Patients with MS should report new or worsened symptoms to their MS team within 3 days of experiencing these symptoms.	Patienten mit MS sollten neue oder sich verschlimmernde Symptome innerhalb von 3 Tagen nach Auftreten ihrem MS Team berichten.	E1. report new symptoms	48
	E2	The MS team should respond within 1 day to a patient with MS reporting an acute deterioration of symptoms.	Das MS Team sollte innerhalb von 1 Tag reagieren, wenn ein Patient eine akute Verschlechterung der Symptome berichtet.	E2. acute deterioration	48
	E3	Patients with MS who experience an acute deterioration of symptoms should be seen by the relevant member of their MS team within 2 days of reporting these symptoms.	Patienten mit MS , die eine akute Verschlechterung der Symptome erleben, sollten innerhalb von 2 Tagen nach Auftreten dieser Symptome von einem entsprechenden MS Team-Mitglied gesehen werden.	E3. referral to MS team	48
