# Supplementary material for: MS brain health quality standards: a survey on the reality in clinical practice in Germany
Source: Neurol Res Pract. 2024 Nov 18;6:59. doi: 10.1186/s42466-024-00333-4 (PMC11571952; doi:10.1186/s42466-024-00333-4)

International consensus on quality standards for brain health-focused care in multiple sclerosis

Results of an expert survey

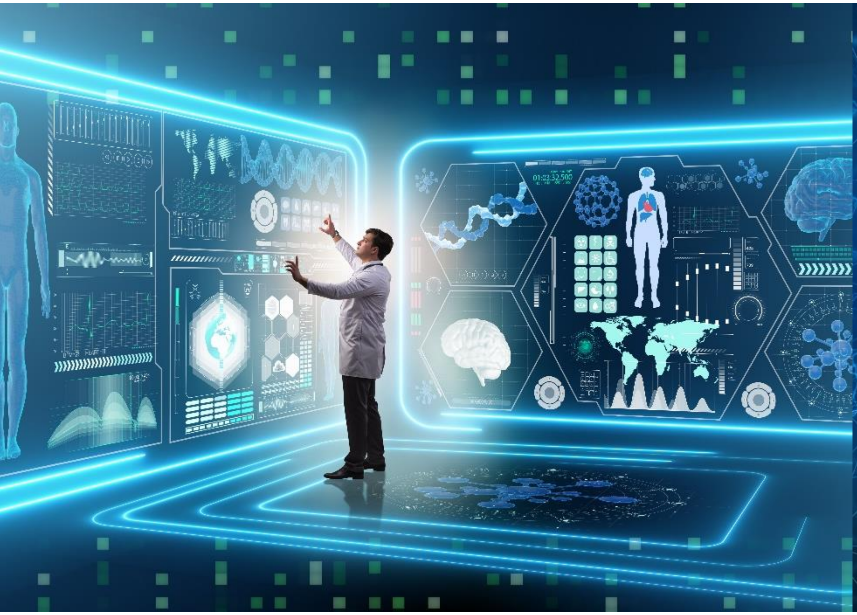
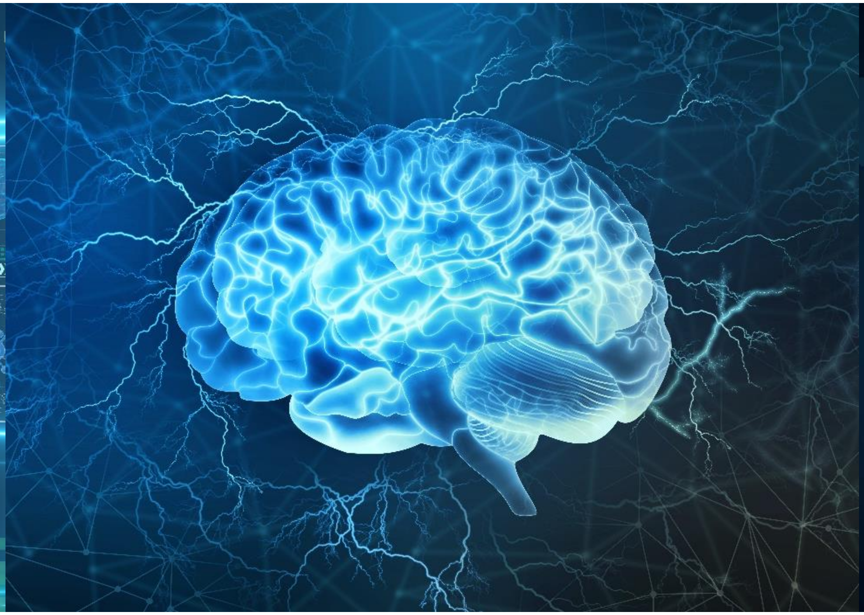
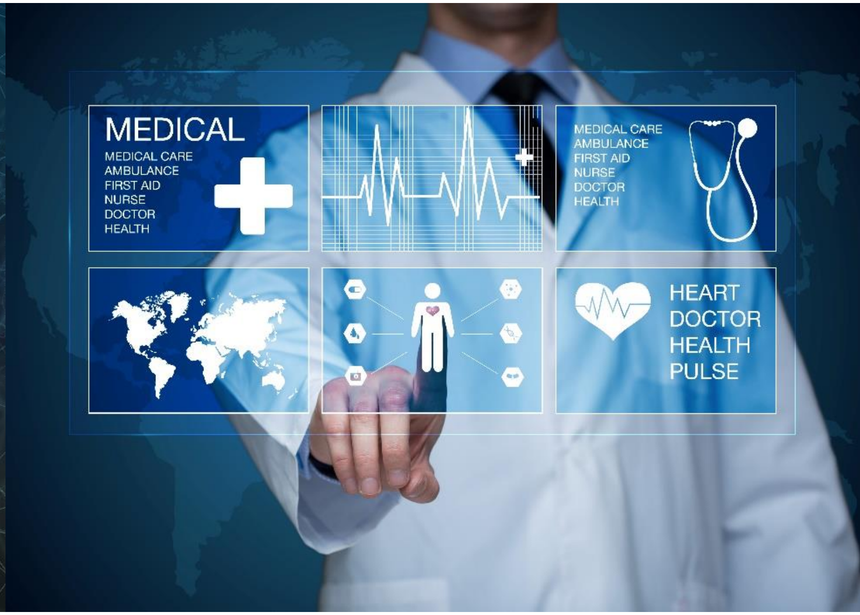

Survey: time-based quality standards of BHI

results

demographics

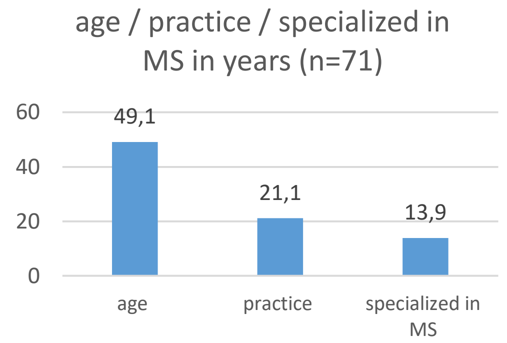

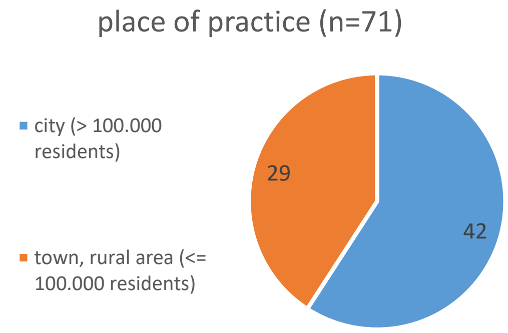

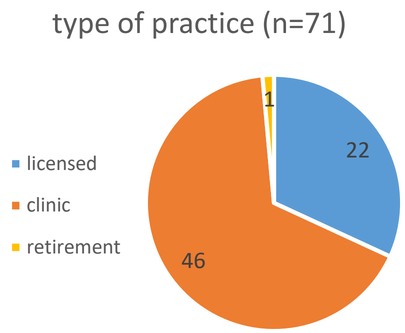

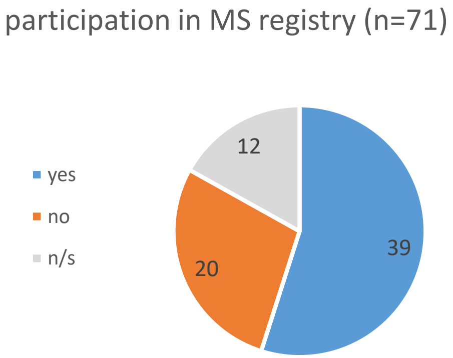

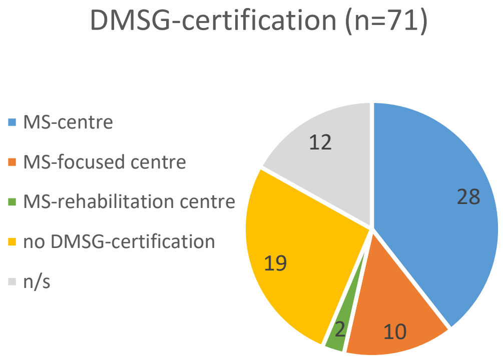

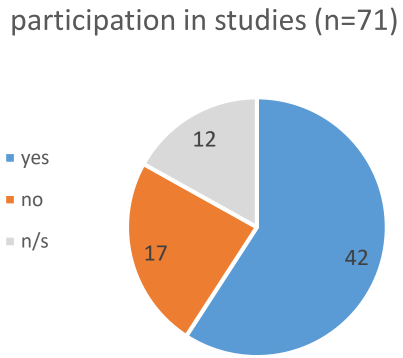

	n=71
**age** (years)	
mean (SD)	49.1 (10.4)
median (range)	49 (29-73)
**duration of practice** (years)	
mean (SD)	21.1 (10.3)
median (range)	13 (2-47)
**duration specialized in MS** (years)	
mean (SD)	13.9 (9.2)
median (range)	20 (0-35)
**place of practice**, n (%)	
city (> 100.000 residents)	42 (59.2%)
town, rural area (<= 100.000 residents)	29 (40.8%)
**type of practice**, n (%)	
licensed	22 (31.0%)
self-employed	17 (23.9%)
medical care center	5 (7.0%)
clinic	46 (64.8%)
outpatient	25 (35.2%)
inpatient	20 (28.2%)
out- and inpatient	1 (1.4%)
retirement	1 (1.4%)
rehabilitation clinic	2 (2.8%)
**participation MS registry**, n (%)	
Yes	39 (54.9%)
No	20 (28.2%)
n/s	12 (16.9%)
**DMSG certification**	
MS-center	28 (39.4%)
MS-focused center	10 (14.1%)
MS-rehabilitation center	2 (2.8%)
no DMSG certification	19 (26.8%)
n/s	12 (16.8%)
**participation in studies**	
yes	42 (59.2%)
no	17 (23.9%)
n/s	12 (16.9%)

Survey: time-based quality standards of BHI

results

A. referral and diagnosis (in sum)

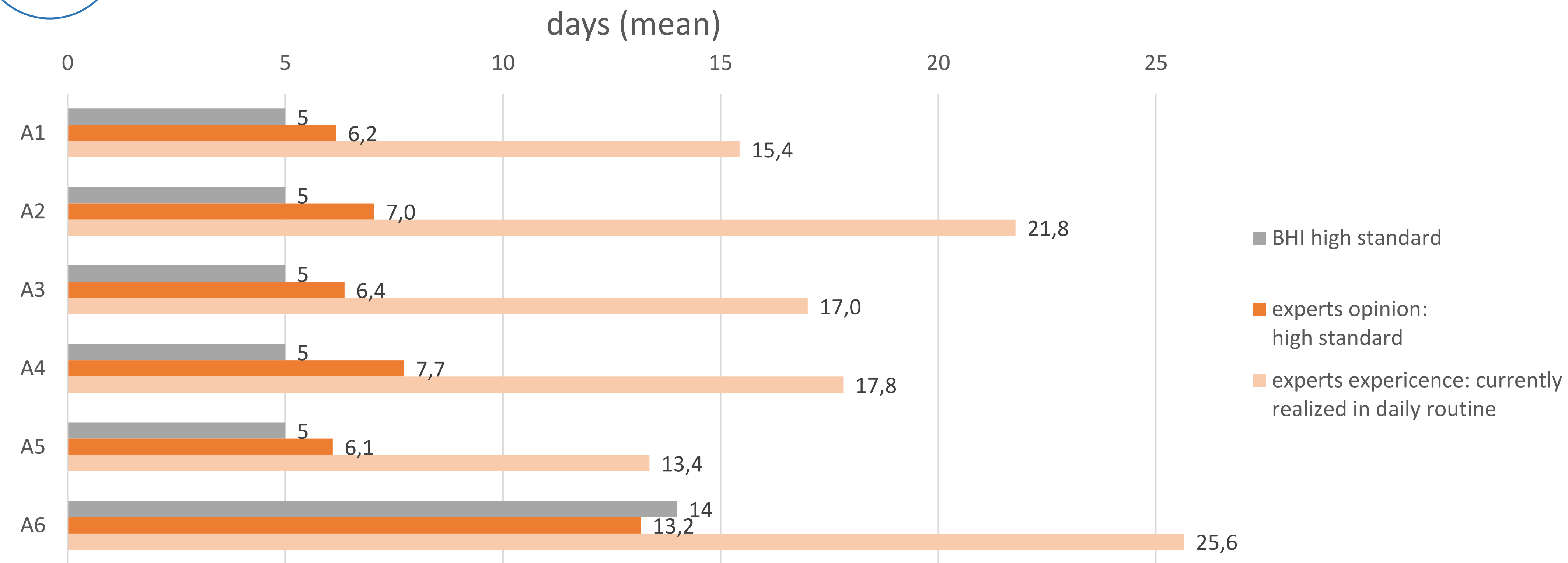

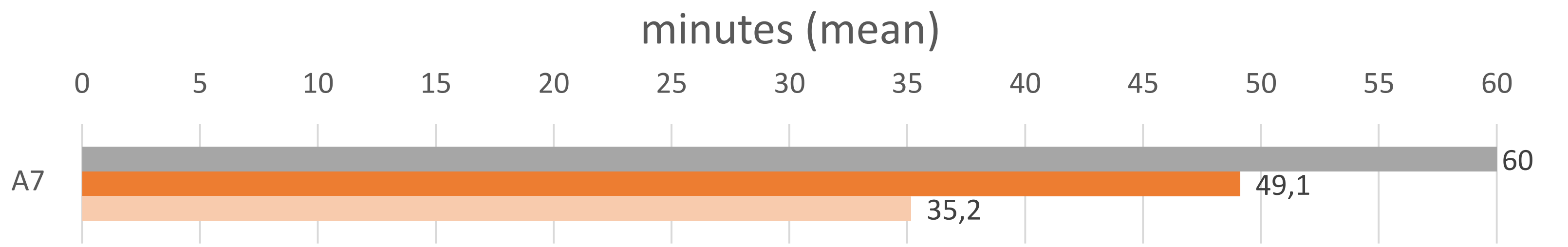

Survey: time-based quality standards of BHI

results

A. referral and diagnosis (in sum)

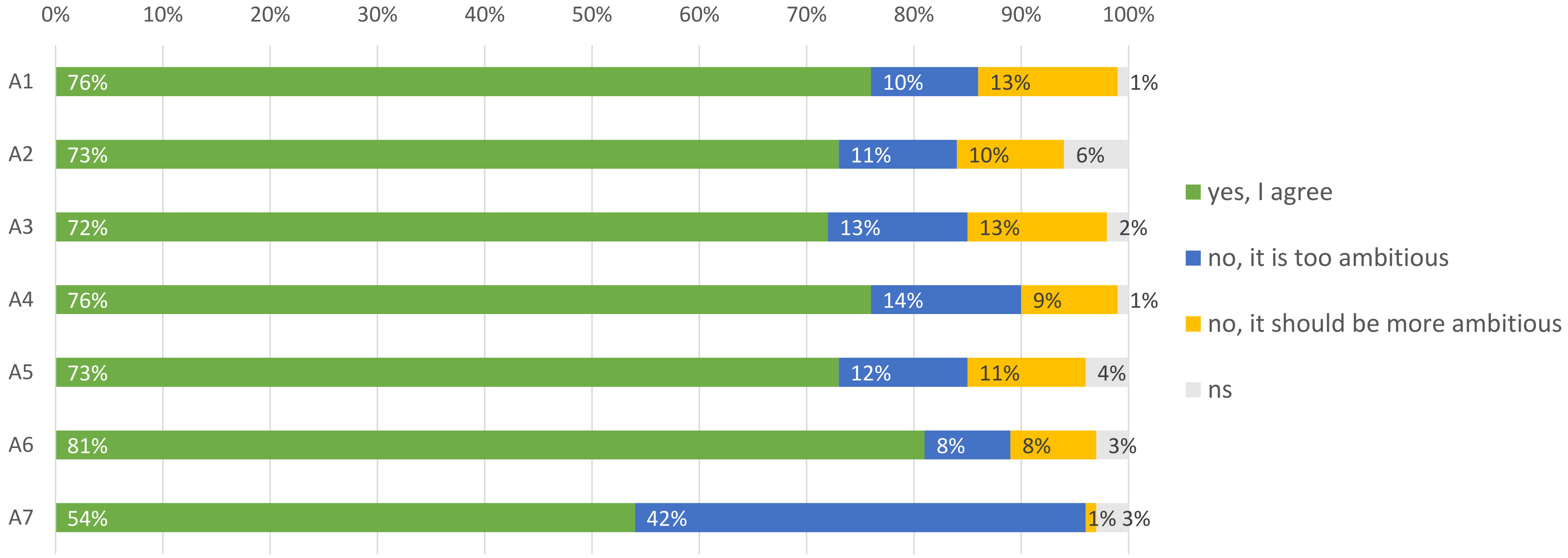

Survey: time-based quality standards of BHI

results

A. referral and diagnosis (1/9)

A1. Anyone experiencing for the first time symptoms that might be related to MS should report them to a healthcare professional within […] days of noticing them. (n=71)

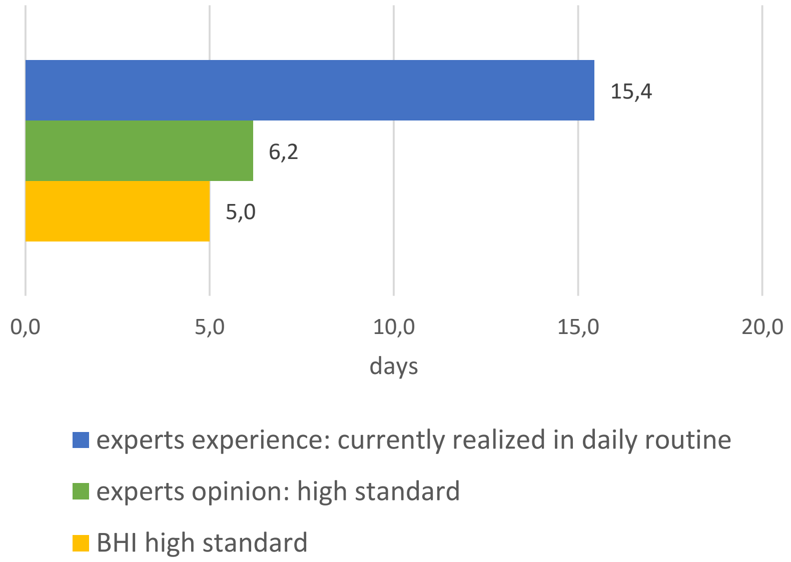

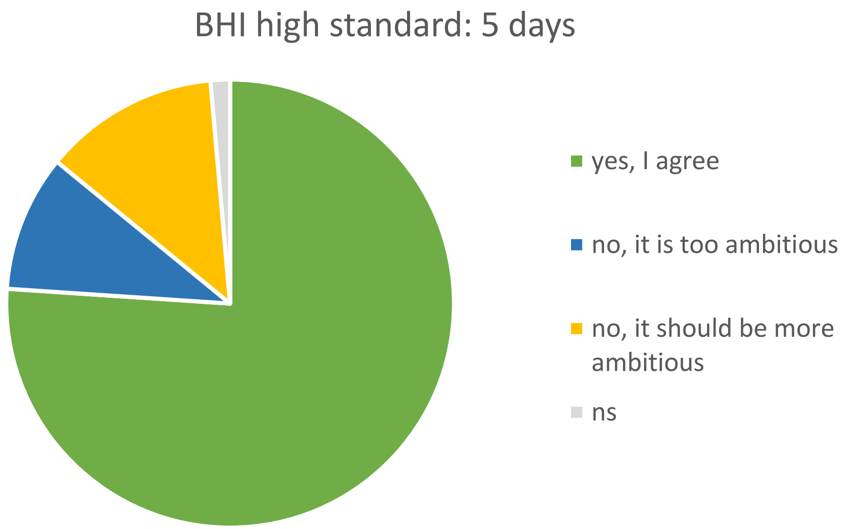

Survey: time-based quality standards of BHI

results

A. referral and diagnosis (1/9)

A1. Anyone experiencing for the first time symptoms that might be related to MS should report them to a healthcare professional within 5 days of noticing them. (n=71)

comments „yes, I agree“
Unless there are signs of paralysis or similarly blatant symptoms
The earlier, the better for the patient
This statement is difficult because the person has not yet been diagnosed with MS and may therefore misclassify the symptoms, not take them seriously or the doctor may misjudge them and thus delay the necessary diagnosis.
depends on the patient and the symptoms as to when he/she reports to the attending physician
although much depends on the symptom. It is treated differently for paresthesia than for hemiparesis or optic neuritis.
However, the time it takes to see a doctor depends heavily on the person affected, shorter for pronounced & disabling symptoms, longer for slowly creeping symptoms.
Initially, the patient will report the symptoms to their GP. Outpatient neurological appointments are only available within 3 months, so the patient is often referred to the clinic by the GP for an initial diagnosis. If the symptoms are apoplectic, the patient is often admitted on the day they first occur or a day later under the suspicion of an apoplectic stroke.

comments „no, it is too ambitious“
No matter how ambitious - it's unrealistic
Does not reflect the reality of care
not realistic in the current care situation and how should the affected person recognize that it is first symptoms of MS

comments „no, it should be more ambitious“
It is desirable to contact a doctor in the event of a new neurological deficit lasting longer than 24 hours
Important DD could be overlooked

Survey: time-based quality standards of BHI

results

A. referral and diagnosis (2/9)

A2. Anyone who reports symptoms that might be related to MS to a healthcare professional should be referred to a neurologist within […] days. (n=71)

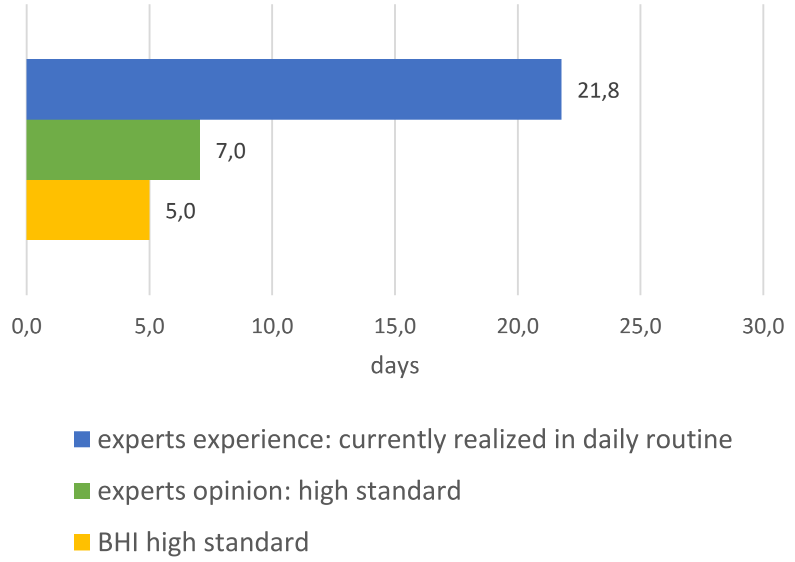

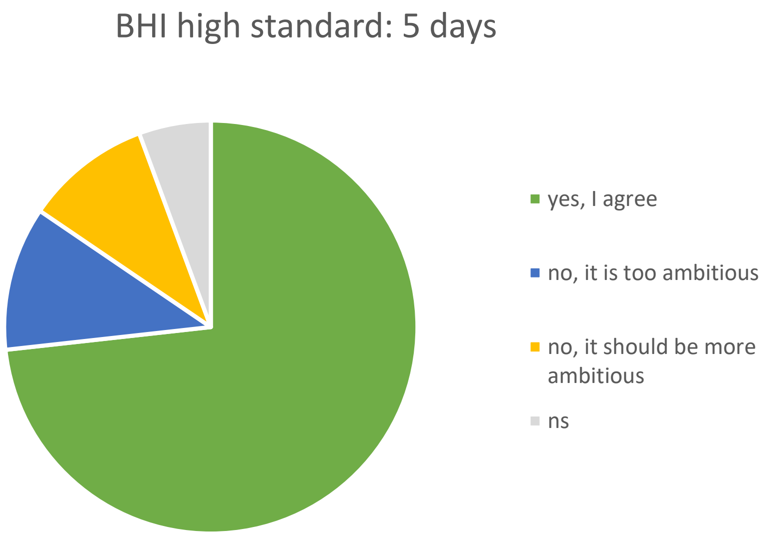

Survey: time-based quality standards of BHI

results

A. referral and diagnosis (2/9)

A2. Anyone who reports symptoms that might be related to MS to a healthcare professional should be referred to a neurologist within 5 days. (n=71)

**comments „yes, I agree"**
but it is not always feasible in reality
but is not feasible in reality
as already mentioned, this depends on the severity of the symptoms
not always feasible in practice
often not feasible in practice
is often not realized due to appointment problems with neurologists. However, severe symptoms often lead to direct hospitalization, even within 1 to 2 days.
As I am not a neurologist in private practice, I cannot currently estimate how long the waiting times are with my colleagues and whether they offer additional appointments for such patients. However, experience shows that patients do not get an outpatient appointment with a neurologist within 5 days and often end up in our clinic. In my MS outpatient clinic, I can only treat patients who are referred by a neurologist in private practice.

**comments „no, it is too ambitious"**
It should be differentiated according to the severity of the symptoms, the more pronounced they are, the sooner action is required.
no matter how ambitious - is unrealistic
Depending on the symptom. I agree in the case of reduced vision, paresis or double vision, but not in the case of purely sensory symptoms, which overwhelm the current healthcare system

**comments „no, it should be more ambitious"**
depending on symptoms (affected system)

Survey: time-based quality standards of BHI

results

A. referral and diagnosis (3/9)

Someone who has symptoms of MS for the first time should primarily present to: Primary Care Physician, General Practitioner) / Neurologist. (n=71)

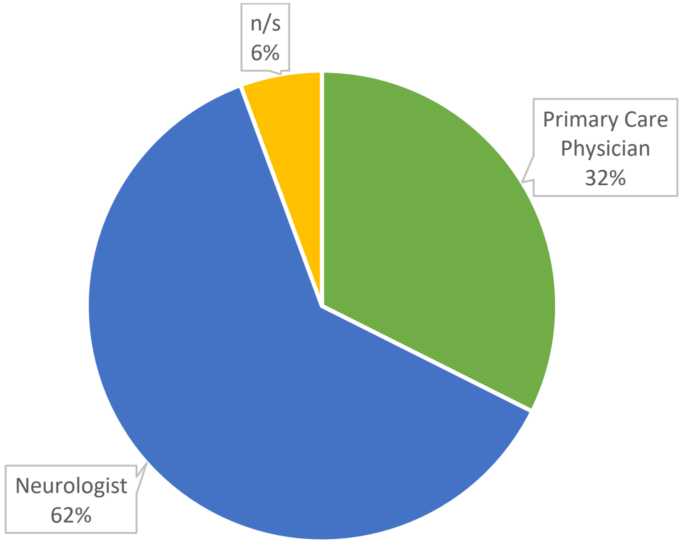

Survey: time-based quality standards of BHI

results | **A. referral and diagnosis (3/9)**

Someone who has symptoms of MS for the first time should primarily present to: Primary Care Physician, General Practioner) / Neurologist. (n=71)

comments „primary care physician“
Control function by GP MUST be retained! Neurologists will otherwise be inundated with mood disorders with a reduction in capacity for MS cases
The specialist cannot assess every new symptom that occurs for the first time; the general practitioner should set the course.
the neurologist is not the first point of contact due to a lack of appointments in most cases
Here too, much depends on the symptom. In many cases, an initial presentation via the emergency room would even be advisable for the first relapse (faster diagnosis and treatment).
faster referral to a neurologist by the GP, if patient calls neurology practices himself, he will often get an appointment too late.
Neurological would be nice, but can only be realized after a delay. The GP can take on the role of presenting the patient promptly either in hospital or as an outpatient neurologist, depending on the symptoms. Outpatient neurological appointments are often only available after a delay.
GP should know the patient, their medical history and personality structure and therefore be able to assess whether the symptoms are "real" or functional. In this case, however, prompt referral to a neurologist, ideally with an MRI.

comments „neurologist“
Unfortunately, this cannot be implemented, neither organizationally nor in terms of patient understanding. Not even all GPs immediately conclude from typical complaints that a neurological examination is necessary.
Emergency room with the involvement of a neurologist or neuropaediatrician
would be desirable, but is not realistic
GP should also be informed
Neurology specialists can better assess the symptoms described and come to a diagnosis more quickly
However, this is not practically feasible.
Should - the reality looks different
directly with the neurologist to avoid delays. Tactically, however, the GP may be the first point of contact because- the patient may not be able to classify their symptoms in terms of specialty- the GP often has a good "connection" to a neurologist and can get appointments quickly.
Should be possible, if necessary after pre-screening by Physician Assistant or similar.
Be realistic

Survey: time-based quality standards of BHI

results

A. referral and diagnosis (4/9)

A3. An initial MRI scan should be performed within […] days of first referral to a neurologist for diagnosis (if not performed earlier). (n=67)

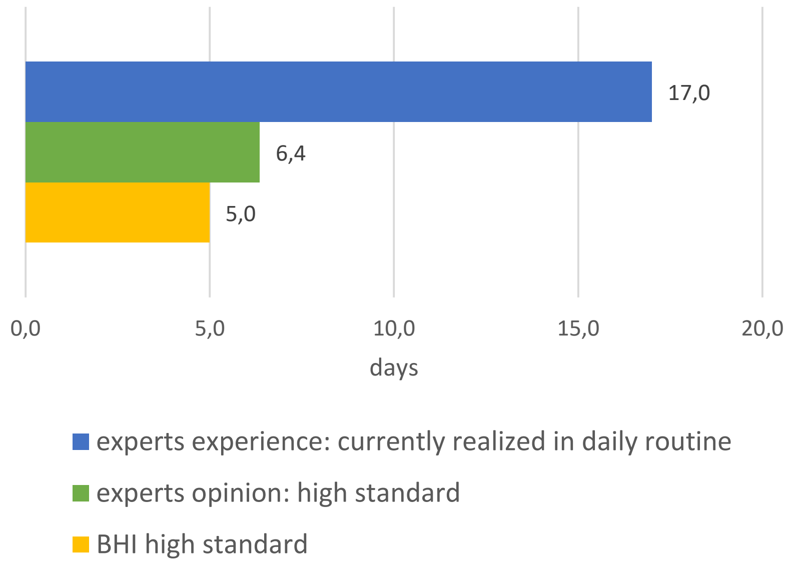

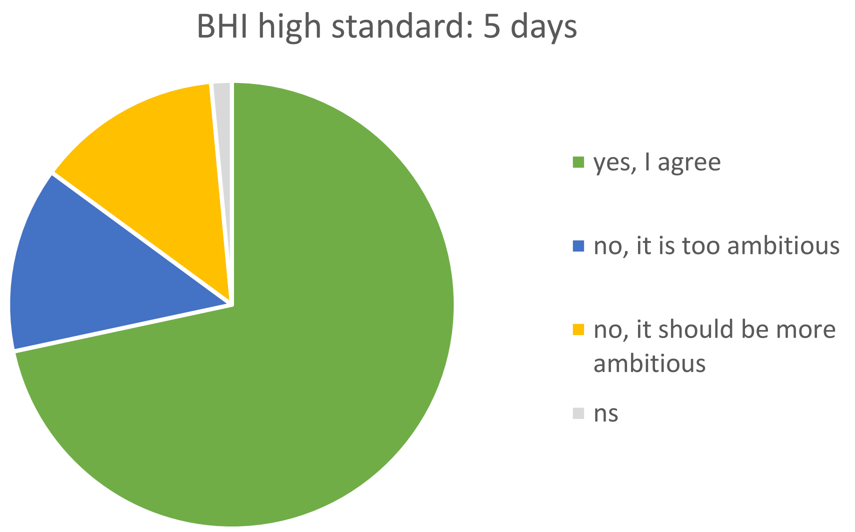

Survey: time-based quality standards of BHI

results

A. referral and diagnosis (4/9)

A3. An initial MRI scan should be performed within 5 days of first referral to a neurologist for diagnosis (if not performed earlier). (n=67)

comments „yes, I agree“
As this is usually not possible on an outpatient basis, I almost always refer patients for initial diagnostics.
Depending on its severity, it should be acted upon.
only works if the attending GP calls the radiology practice directly, otherwise the waiting times are significantly longer
MRI availability has improved and can realistically be implemented if the patient is flexible in terms of travel and time.
A rapid MRI is desirable to rule out differential diagnoses.

comments „no, it should be more ambitious“
In a hospital outpatient clinic, a cMRI is possible on the first day of presentation, but not in the practices, of course

Survey: time-based quality standards of BHI

results

A. referral and diagnosis (5/9)

An initial MRI should be performed BEFORE the initial presentation to the neurologist. (n=65)

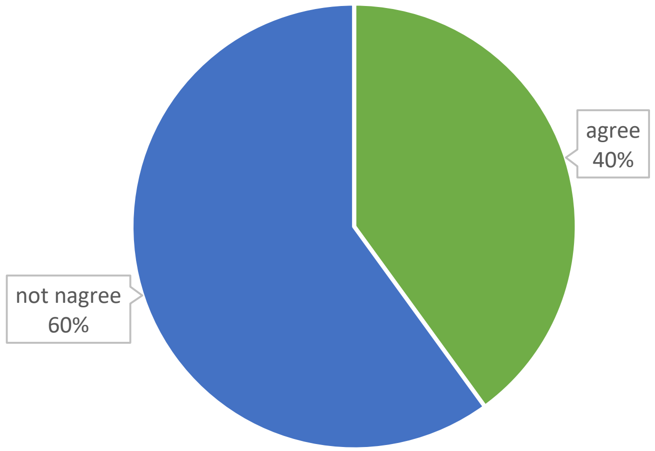

Survey: time-based quality standards of BHI

results

A. referral and diagnosis (6/9)

A4. The MS team should complete a diagnostic workup for MS within […] days of referral to a neurologist. (n=66)

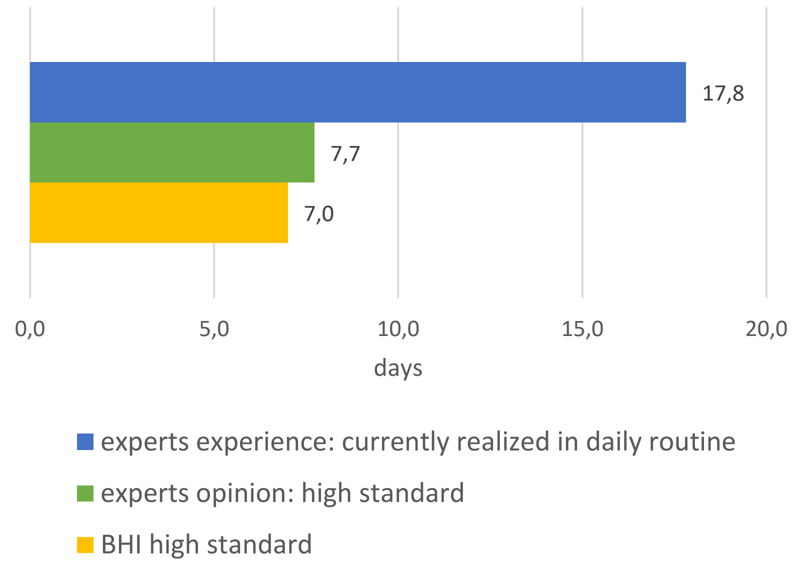

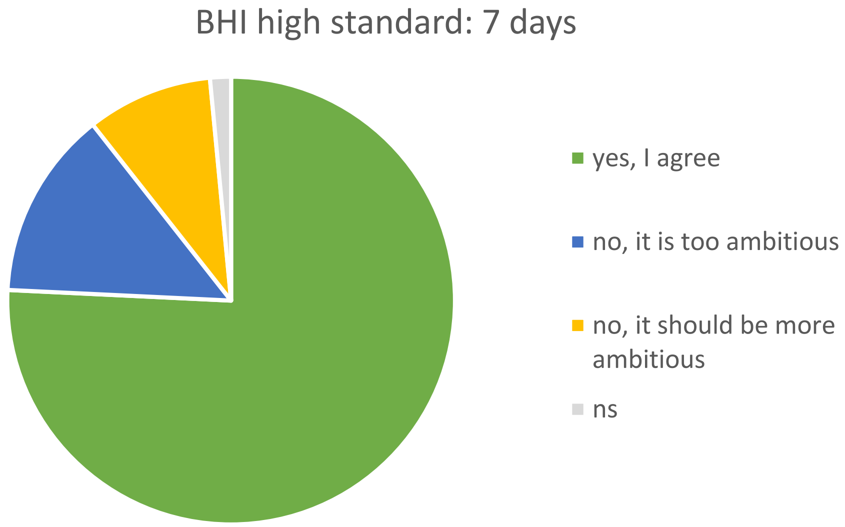

Survey: time-based quality standards of BHI

results

A. referral and diagnosis (6/9)

A4. The MS team should complete a diagnostic workup for MS within 7 days of referral to a neurologist. (n=66)

comments „yes, I agree“
but is probably difficult to implement in reality
Not feasible simply because of the waiting times for an MRI.
A complete diagnosis also includes a CSF examination after the clinical and MRI examination, the results of which are available within 1-2 days (individual infection titers may take longer).
usually takes longer before findings are obtained
my reason for "no" in the previous question (first MRI): the neurologist should see the patient and then determine the extent of the MRI (e.g. plus spinal axis)
As I work in a clinic, I can't say how long the colleagues in private practice need for the diagnosis. In our region, almost all patients are referred to the clinic by the GP or neurologist for initial diagnosis (incl. LP etc.). Here we need 3 days for the diagnosis.

comments „no, it is too ambitious“
Some lab results unfortunately take more than 7 days
Spinal MRI and LP (+ electrophysiology if necessary) within 7 weeks logistically complicated.

Survey: time-based quality standards of BHI

results

A. referral and diagnosis (7/9)

A5. The results from a diagnostic workup for MS should be discussed within […] days of completion, during an appointment with the patient. (n=66)

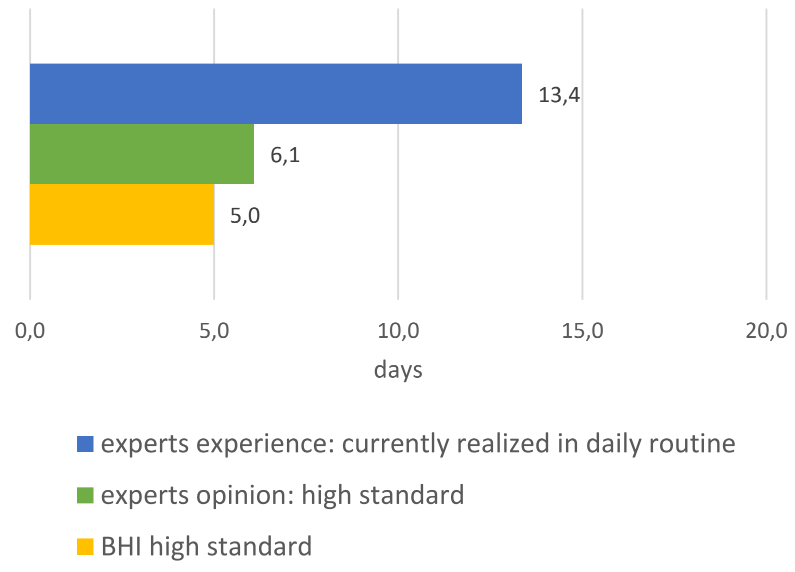

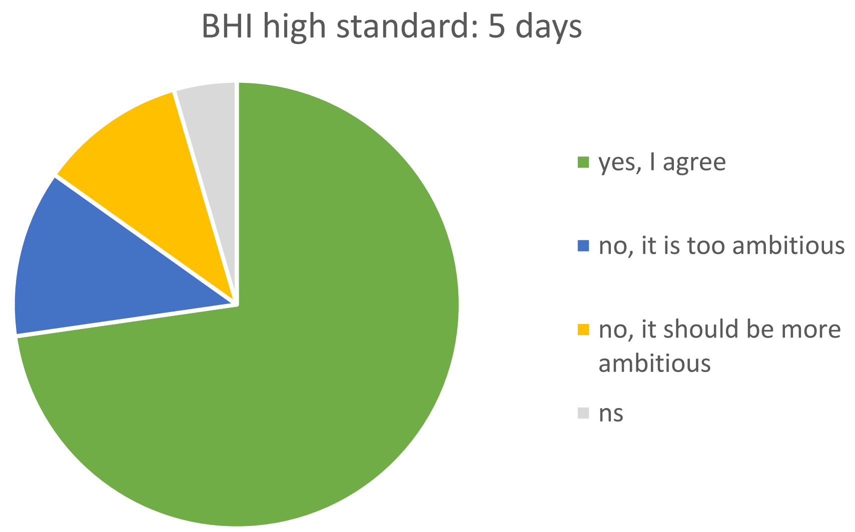

Survey: time-based quality standards of BHI

results

A. referral and diagnosis (7/9)

A5. The results from a diagnostic workup for MS should be discussed within 5 days of completion, during an appointment with the patient. (n=66)

comments „yes, I agree"
Would be ideal, but this is not always feasible in practice. However, if pathological findings are made, the evaluation with the patient always takes place promptly (within a few days)
if pathological and relevant to therapy
not always feasible in practice
the period may be slightly longer (10 days) if the symptoms are less pronounced.
The suspected diagnosis is discussed in the clinic on the day of discharge after a 3-day inpatient stay.

comments „no, it is too ambitious"
The values are generally not available in such a timely manner.
For free appointments within 5 days, more MS specialists and better financial conditions with health insurance companies and authorities would be needed

comments „no, it should be more ambitious"
This should be done within 24 hours for inpatients and within 7 days for outpatients
Findings should be discussed with the patient immediately

Survey: time-based quality standards of BHI

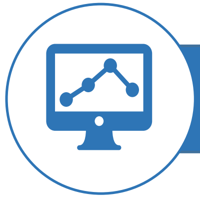

results

A. referral and diagnosis (8/9)

A6. An accurate diagnosis of (uncomplicated) MS should be made and communicated to the patient within […] weeks of their referral to a neurologist. (n=65)

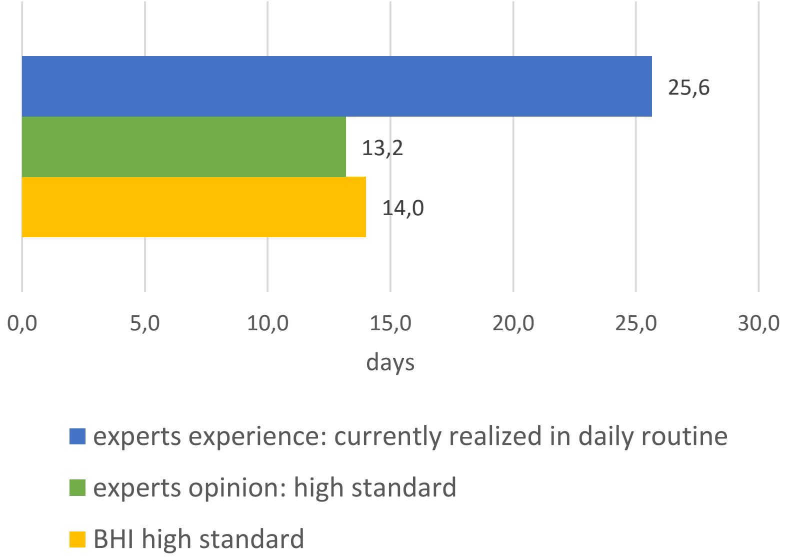

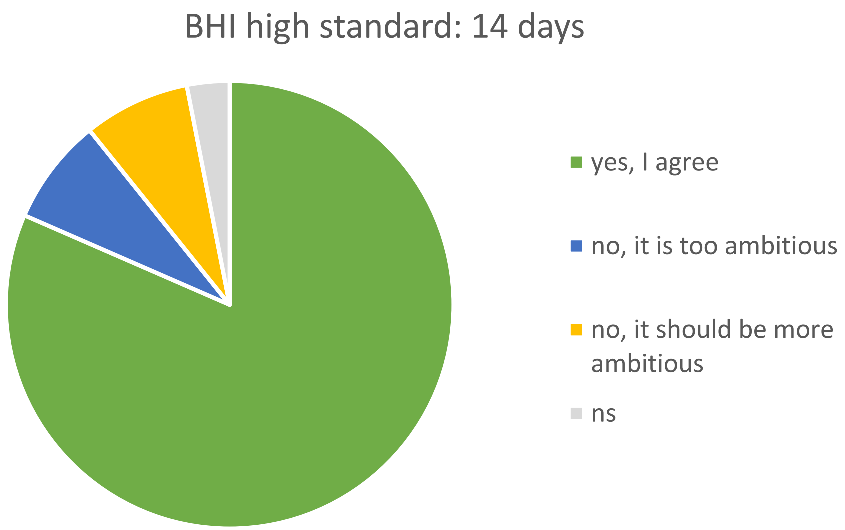

Survey: time-based quality standards of BHI

results

A. referral and diagnosis (8/9)

A6. An accurate diagnosis of (uncomplicated) MS should be made and communicated to the patient within 2 weeks of their referral to a neurologist. (n=65)

comments „yes, I agree“
However, if the results of the diagnostics are to be communicated only 5 days after they have been carried out, hardly any patient will want to go home without a diagnosis and see a neurologist again 9 days later.

comments „no, it should be more ambitious“
For inpatients this should be 1d after all results are available, for outpatients this should be after max. 7d.
aim for shorter periods, as a treatment decision should also be made and this can often only be implemented after 2-3 more weeks.

Survey: time-based quality standards of BHI

results

A. referral and diagnosis (9/9)

A7. Following MS diagnosis, patients should be offered an initial appointment of at least […] hour to discuss the implications of the diagnosis. (n=65)

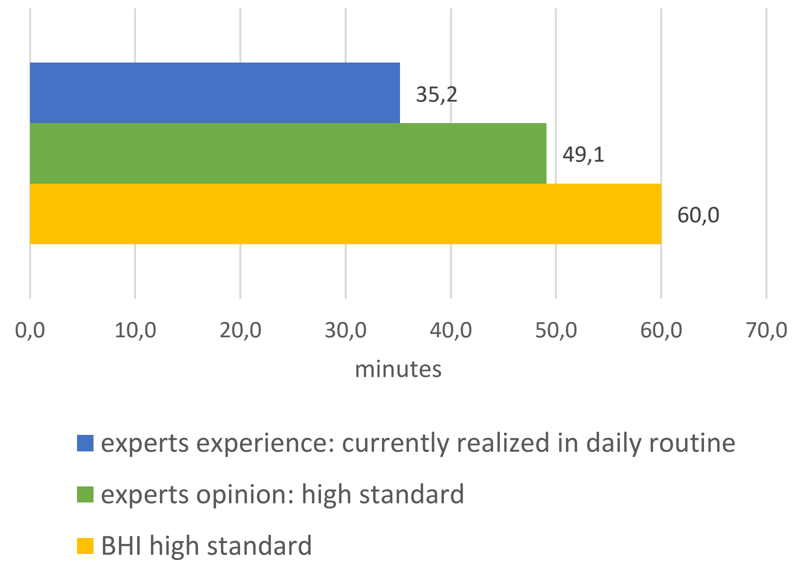

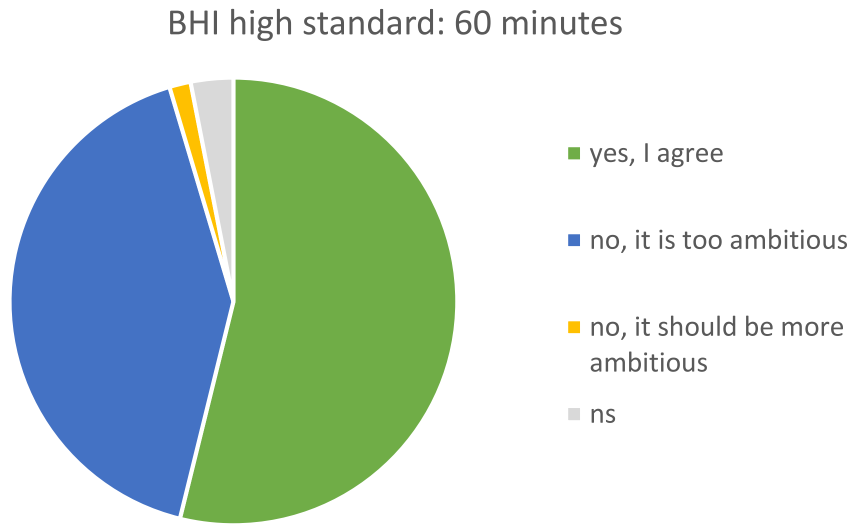

Survey: time-based quality standards of BHI

results

A. referral and diagnosis (9/9)

A7. Following MS diagnosis, patients should be offered an initial appointment of at least 1 hour to discuss the implications of the diagnosis. (n=65)

comments „yes, I agree“
but is not feasible in reality
Here, too, financing is difficult in Germany
A length of more than 60 minutes would certainly be too demanding for the patient. A 2nd appointment approx. 10 days later is recommended to clarify the questions that have arisen from the 1st consultation.

comments „no, it is too ambitious“
1. 60min cannot be realized; 2. longer than 45min does not hold the patient's capacity; 3. it is better to allocate call capacities to follow-up calls
Perhaps desirable, but not feasible in everyday practice
Information should be complete. A first appointment of this duration is too much for the patient. It is better to have 2-3 appointments in quick succession. A total duration of 60 minutes is realistic
We make several appointments, also with partners or relatives
If the patient has already received information in the meantime, 15-20 minutes will certainly suffice. is certainly enough, especially as a second appointment is usually required to finalize the therapy agreement.
so much time that would be nice
rather offer 2 interviews for diagnosis and therapy
is desirable, but does not correspond to the reality of supply
45 minutes is sufficient, otherwise the patient is overloaded with information.
This is partly information overload, better repeated appointments
Sometimes few questions at first, I personally prefer to offer a follow-up appointment after 3-4 weeks.
In the MS outpatient clinic, I definitely take up to 45 minutes. In outpatient neurology, I think that's utopian given the current volume of patients.

Survey: time-based quality standards of BHI

results

B. priorities following diagnosis (in sum)

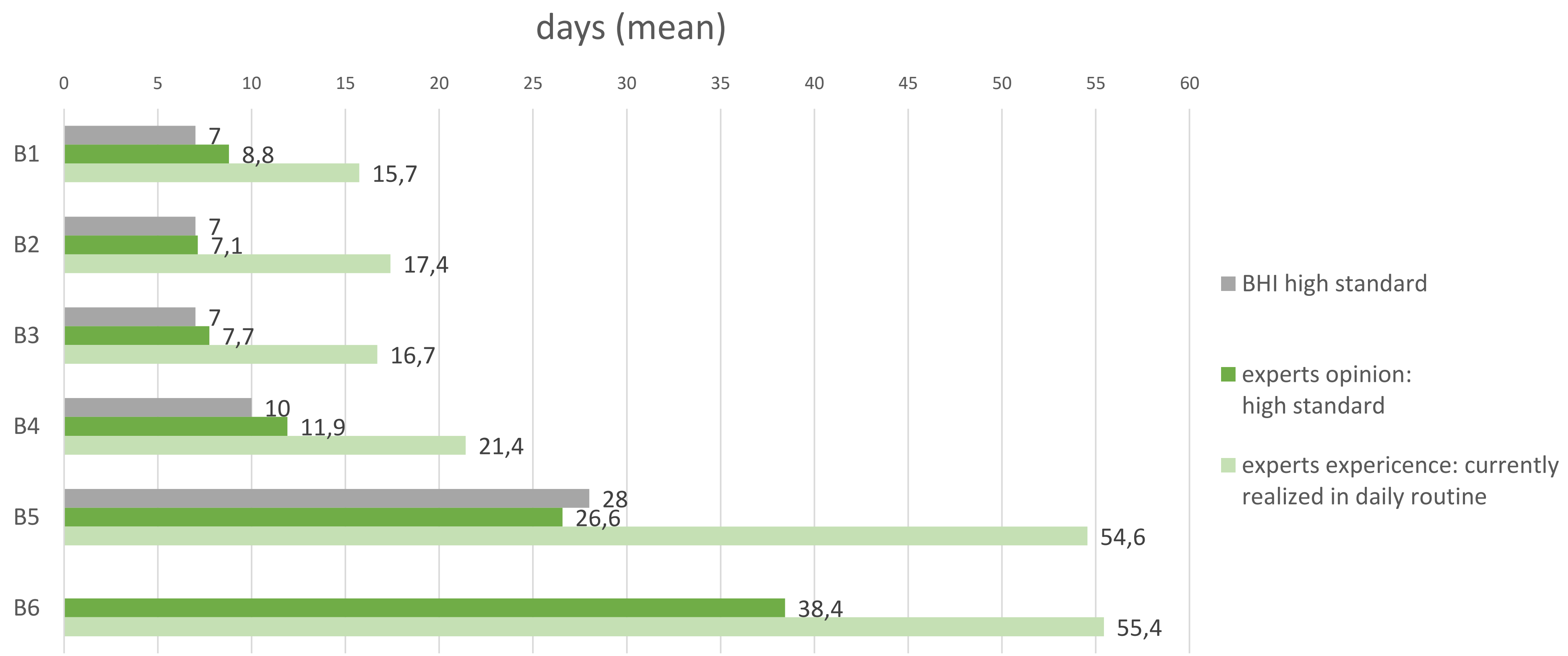

Survey: time-based quality standards of BHI

results

B. priorities following diagnosis (in sum)

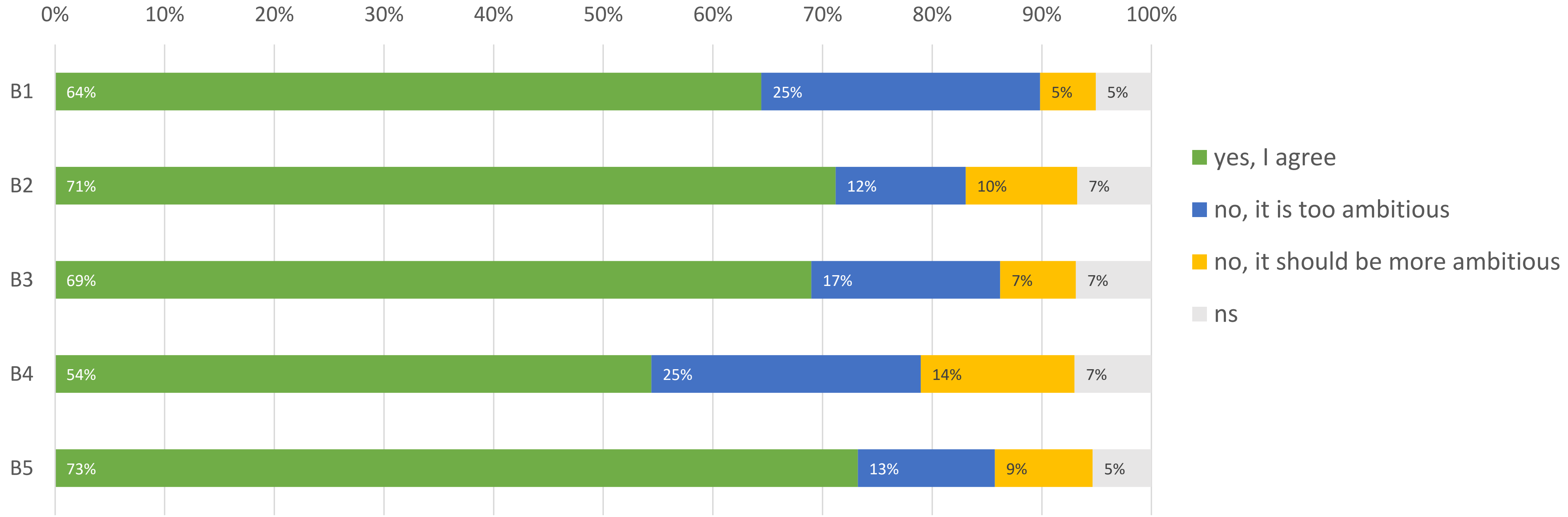

Survey: time-based quality standards of BHI

results

B. priorities following diagnosis (1/9)

B1. The MS team should discuss the aims of treatment with each patient within [...] days of MS diagnosis. (n=59)

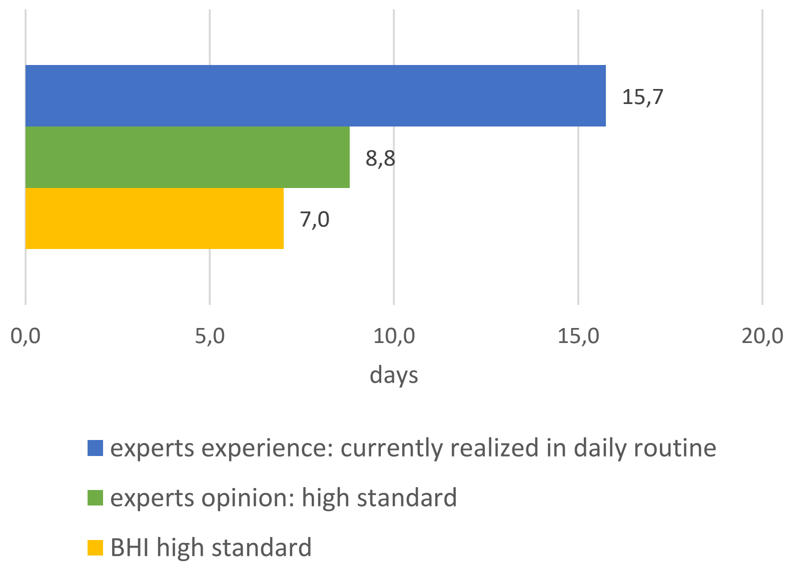

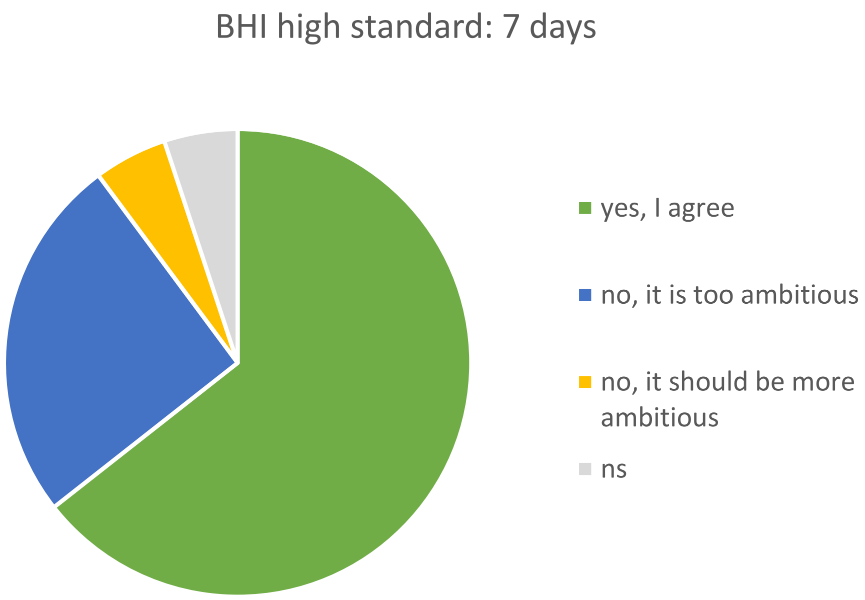

Survey: time-based quality standards of BHI

results

B. priorities following diagnosis (1/9)

B1. The MS team should discuss the aims of treatment with each patient within 7 days of MS diagnosis. (n=59)

**comments „no, it is too ambitious“**
Patients usually need longer to come to terms with the diagnosis and decide on a treatment option.
In many cases, this is not even possible because the patient only listens to the goals at the latest 7 days after the MS explanation and then "nods", but does not yet have / cannot have their own opinion. The discussion of goals should be scheduled individually, depending on the patient's understanding of the disease, how affected they are, etc.

**comments „no, it should be more ambitious“**
depends on the patient with regard to clinic processing
Therapy discussions always in conjunction with the diagnosis notification

Survey: time-based quality standards of BHI

results

B. priorities following diagnosis (2/9)

B2. The MS team should assess within […] days of an MS diagnosis whether the patient is eligible for treatment with a suitable DMT. (n=59)

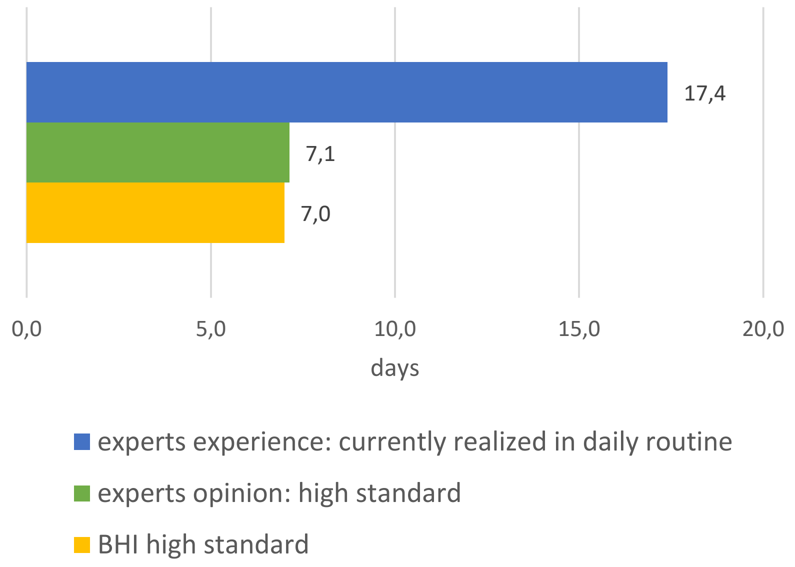

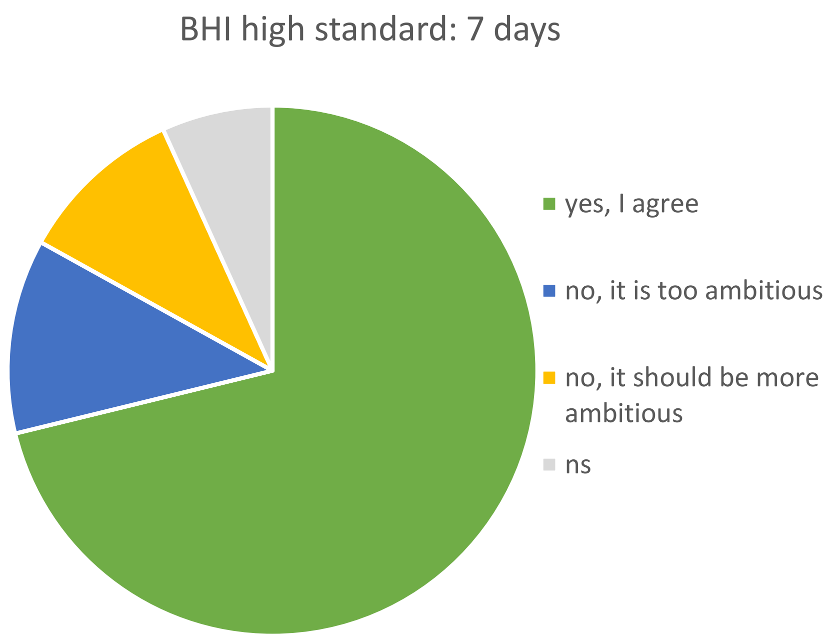

Survey: time-based quality standards of BHI

results

B. priorities following diagnosis (2/9)

B2. The MS team should assess within 7 days of an MS diagnosis whether the patient is eligible for treatment with a suitable DMT. (n=59)

comments „no, it is too ambitious“
In my opinion, this would overwhelm most patients. After all, it is a decision that usually affects many aspects of life planning: partnership, desire to have children, career ... Having to make what is probably a very far-reaching decision within a week can also overwhelm patients, and possibly also the team, which may not even have all the necessary data available at this point. It is often a case of patients who knew about drug therapy "at best" from their parents, but were completely healthy and drug-free themselves. In my opinion, adherence improves if patients are given sufficient time to make a participatory decision.

Kommentare „nein, das sollte ambitionierter sein“
Diagnosis and therapy consultation should take place together

Survey: time-based quality standards of BHI

results

B. priorities following diagnosis (3/9)

B3. The MS team should discuss the pros and cons of early treatment with a DMT with each patient within […] days of diagnosis. (n=58)

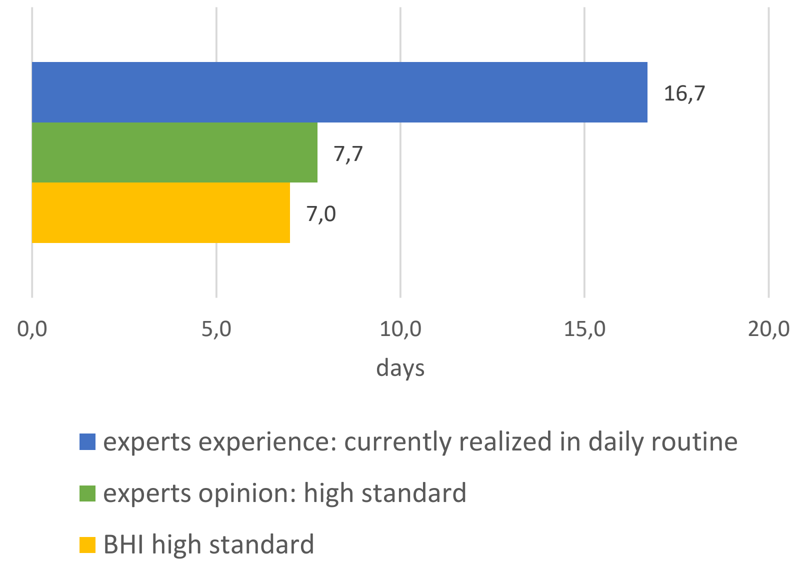

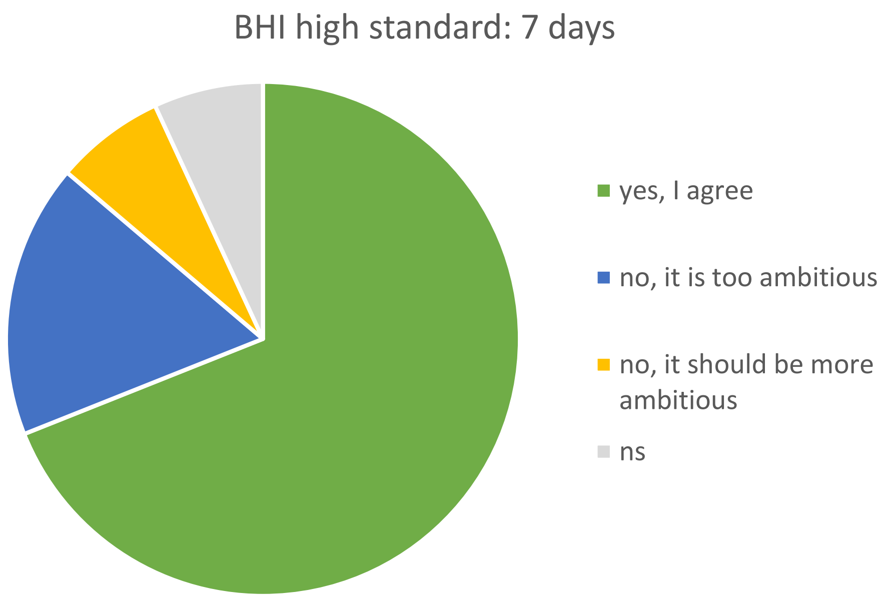

Survey: time-based quality standards of BHI

results

B. priorities following diagnosis (3/9)

B3. The MS team should discuss the pros and cons of early treatment with a DMT with each patient within 7 days of diagnosis. (n=58)

comments „yes, I agree“
Frequent lack of time and late appointments extend the period

comments „no, it is too ambitious“
Patients usually take longer to consent to immunotherapy and to decide when it should be given.
This will not be possible for many patients. It is a completely foreign conceptual world for most patients. I have always decided very quickly on 2 to 3 therapies myself, then given the patients the relevant KKNMS information sheets and met with them again after about 3 weeks to answer questions and then, if necessary, to decide on one of the medications. However, some patients still need time to accept a therapy.

comments „no, it should be more ambitious“
Diagnosis and therapy consultation should take place together

Survey: time-based quality standards of BHI

results

B. priorities following diagnosis (4/9)

B4. The importance of a brain-healthy lifestyle should be discussed with each patient with MS within […] days of diagnosis. (n=57)

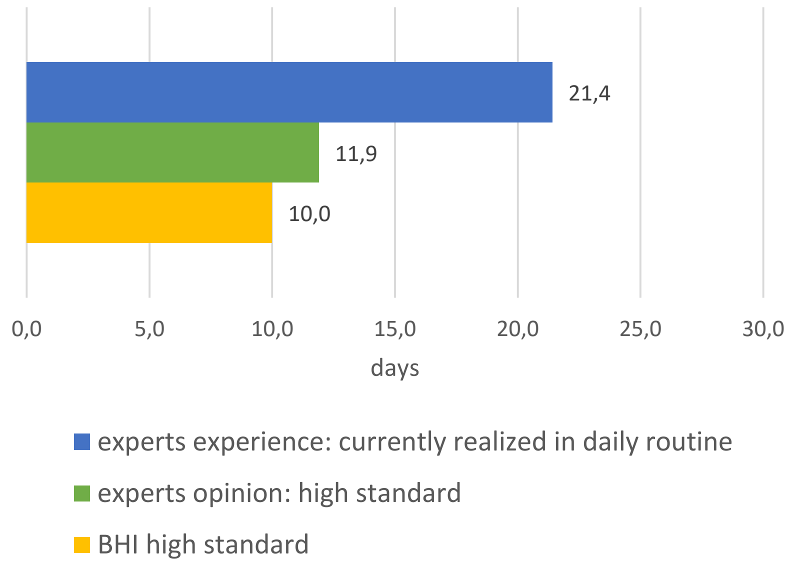

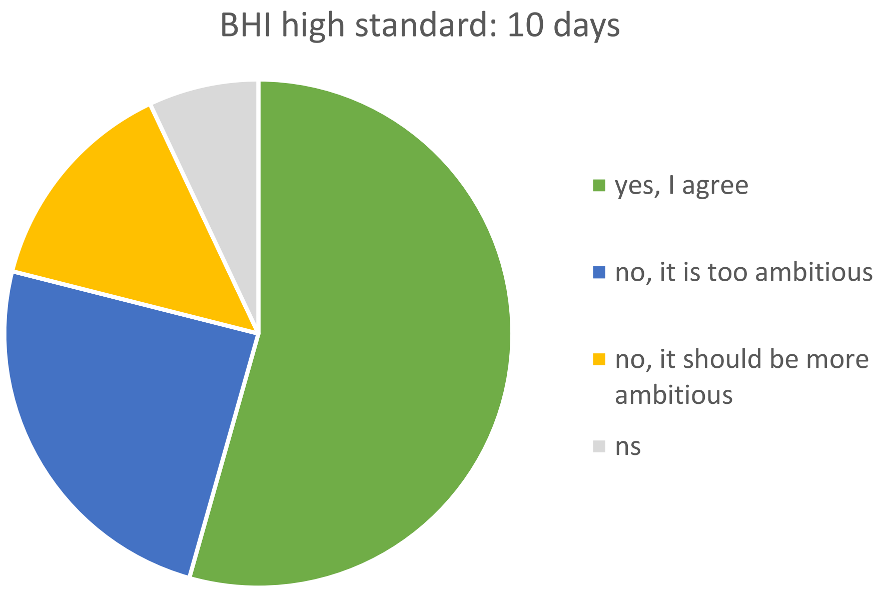

Survey: time-based quality standards of BHI

results

B. priorities following diagnosis (4/9)

B4. The importance of a brain-healthy lifestyle should be discussed with each patient with MS within 10 days of diagnosis. (n=57)

comments „yes, I agree“
Usually discussed at the same time as therapy recommendations

comments „no, it should be more ambitious“
This can be discussed when making a diagnosis

comments „no, it is too ambitious“
The information on lifestyle is important and should be provided initially, whether this is done after 10 or 14 d is irrelevant.
this is again an excessive demand on the patients; they should, within approx. 4 weeks after the onset of symptoms of the disease- have some possibly frightening examinations carried out, - come to terms with the diagnosis of MS, - decide on a very long-term therapy with unknown tolerances and AEs, - possibly change their family planning (which also affects other family members) and - take care of their lifestyle, i.e. usually restrict themselves.This is quite a lot to ask of people who have often not had to deal with illnesses before (even if it is intended to benefit their health).
Not the most important topics, it is better to start a disease-modifying therapy quickly, there is already enough to talk about here, this topic can be discussed as a secondary issue
Should be discussed very cautiously, hardly any evidence
As already mentioned, I prefer follow-up appointments

Survey: time-based quality standards of BHI

results

B. priorities following diagnosis (5/9)

Do you discuss the importance of a "brain healthy" lifestyle with your patients? (n=54)

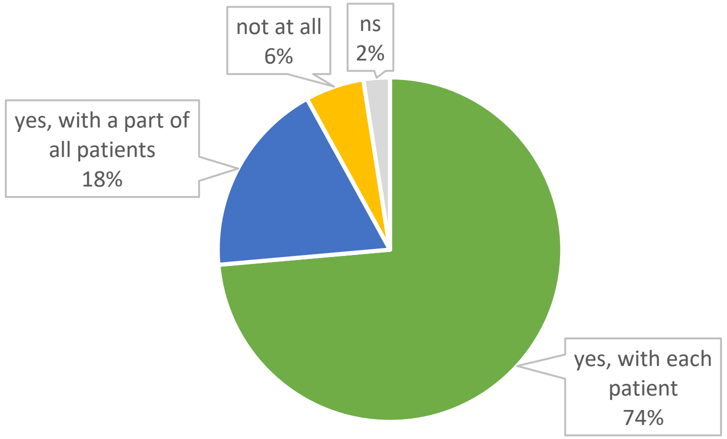

**comments „yes, with each patient“**
often limited in terms of time due to higher workload but always mentioned.
Yes, I discuss the lifestyle with the patient, but not usually within the first few weeks after diagnosis. If you give them a bit of time, I think it's easier to make the change.
There are also so-called over-fulfillers, but I tend to be more cautious here

**comments „no, not at all“**
no evidence

Survey: time-based quality standards of BHI

results

B. priorities following diagnosis (6/9)

B5. Patients with MS who need additional support to make lifestyle modifications, beyond that offered by the MS team, should be referred to appropriate services within [...] weeks of diagnosis. (n=56)

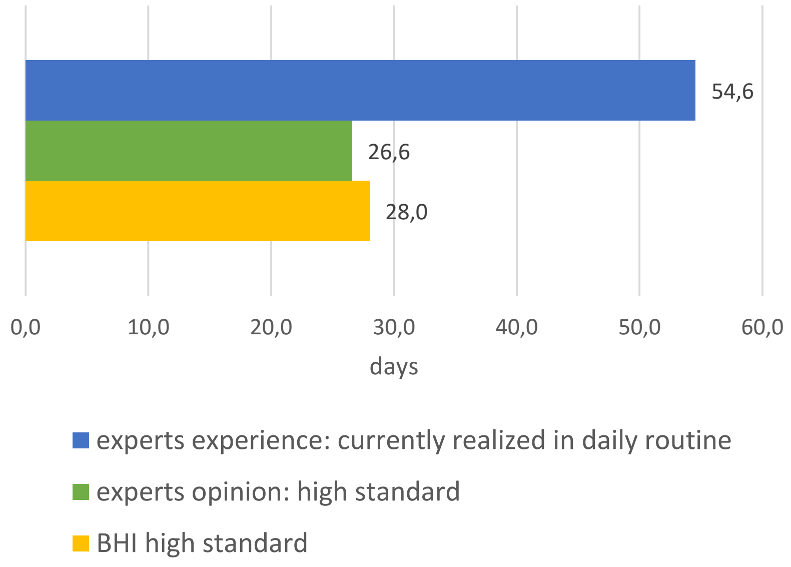

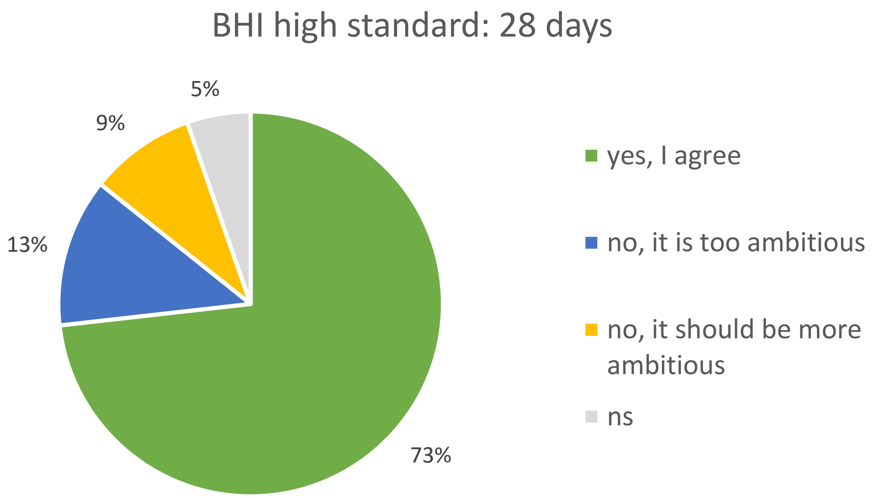

Survey: time-based quality standards of BHI

results

B. priorities following diagnosis (6/9)

B5. Patients with MS who need additional support to make lifestyle modifications, beyond that offered by the MS team, should be referred to appropriate services within 4 weeks of diagnosis. (n=56)

comments „yes, I agree“
Who is the respective service provider?
What is meant by service provider?; other specialist practices, rehabilitation ??, advice from health insurance company ?

comments „no, it is too ambitious“
Not realistic
no, in my opinion this is too fast, see my previous comments (yes, I discuss the lifestyle with the patient, but usually not within the first few weeks after diagnosis. If you give them a bit of time, I think the change will be more successful).

Survey: time-based quality standards of BHI

results

B. priorities following diagnosis (7/9)

Do you refer your patients who need additional lifestyle modification support to appropriate service providers after MS diagnosis? (n=53)

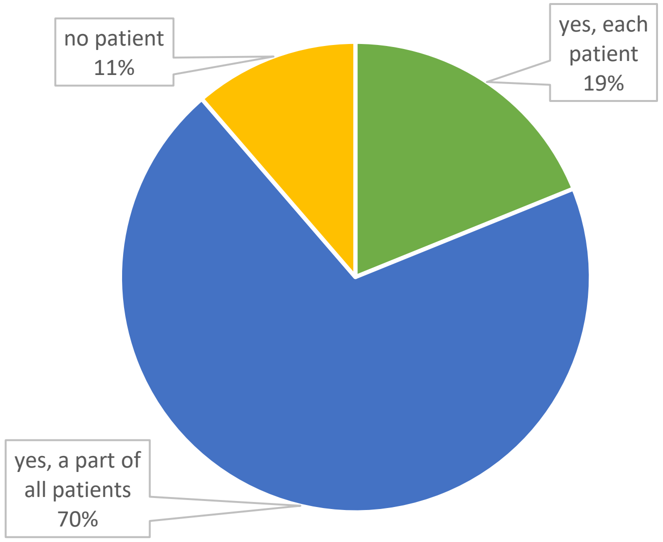

comments „yes, each patient“
Yes, for everyone who needs it.

comments „yes, a part of all patients“
Who is the one?
Not everyone wants to change their lifestyle despite being informed
Rather rare

comments „no patient“
No sufficient infrastructure available

Survey: time-based quality standards of BHI

results

B. priorities following diagnosis (8/9)

B6. Cognitive screening should be offered to all patients with MS within [...] days of diagnosis. (n=53)

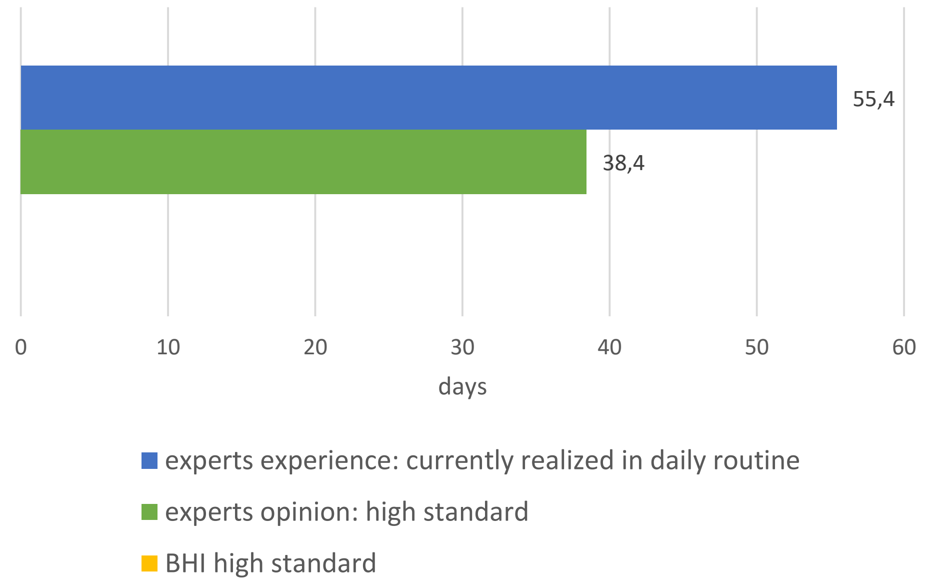

No optimal treatment standard specified

Survey: time-based quality standards of BHI

results

B. priorities following diagnosis (8/9)

B6. Cognitive screening should be offered to all patients with MS within [no high standard] days of diagnosis.

(n=53) - No optimal treatment standard specified

comments
Cognition does not include fatigue, which would be particularly important. The question is formulated too generally and assumes THAT it should be offered to everyone. Therefore, it cannot be answered if - like me - you don't think it should be offered to everyone.
Not too early, because uncertainty as a result of reporting a diagnosis can lead to a false "bad" result.
The idea is right, but not feasible. There are very few (neuro)psychologists who do diagnostics at all.
Baseline values should be collected quickly
All patients should undergo cognitive and fatigue screening within 3 months if possible.
standardized at the time of diagnosis, you have initial data in the course
Screening at the time of diagnosis
Unfortunately, only periods of up to 100 days can be selected in the question. I would have set within 6 months if possible
Not yet established in routine use
Cognitive screening should initially only be carried out in patients with obvious and impairing symptoms. In my opinion, it does not make sense to screen all patients as long as we do not have any really good therapy suggestions. Screening should also be carried out at some distance from the measures mentioned so far.
Would be important though!
Cognitive impairment is an issue in the course of the disease, specific screening at diagnosis is not carried out for every patient, depending on the clinical impression. Impression, everyday problems and cardiac load
Unfortunately not possible due to lack of support from a psychologist
As early as possible in order to be able to plan discussions and resources

Survey: time-based quality standards of BHI

results

B. priorities following diagnosis (9/9)

Do you offer cognitive screening to your patients after MS diagnosis? (n=53)

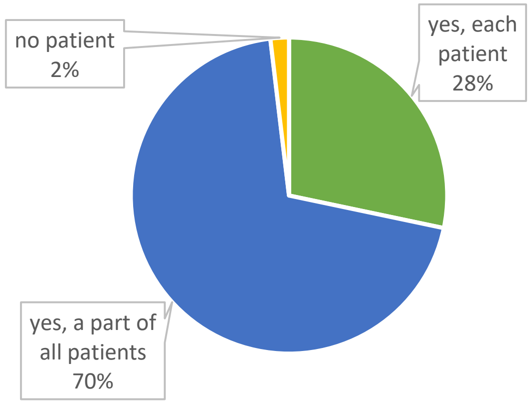

comments „yes, each patient“
rejected by some of the patients
Only SDMT/FSMC, more extensive neuropsychological testing is not feasible in everyday life

comments „yes, a part of all patients“
if there are indications of a cognitive disorder
In the form of a referral to a psychologist/neuropsychologist
more feasible in larger centers. Often rejected by patients.
This is often not possible in practice and the number of suitable neuropsychologists in Germany is still too low.
In-house infrastructure inadequate and not sufficiently sensitized

Survey: time-based quality standards of BHI

results

C. routine monitoring and support (in sum)

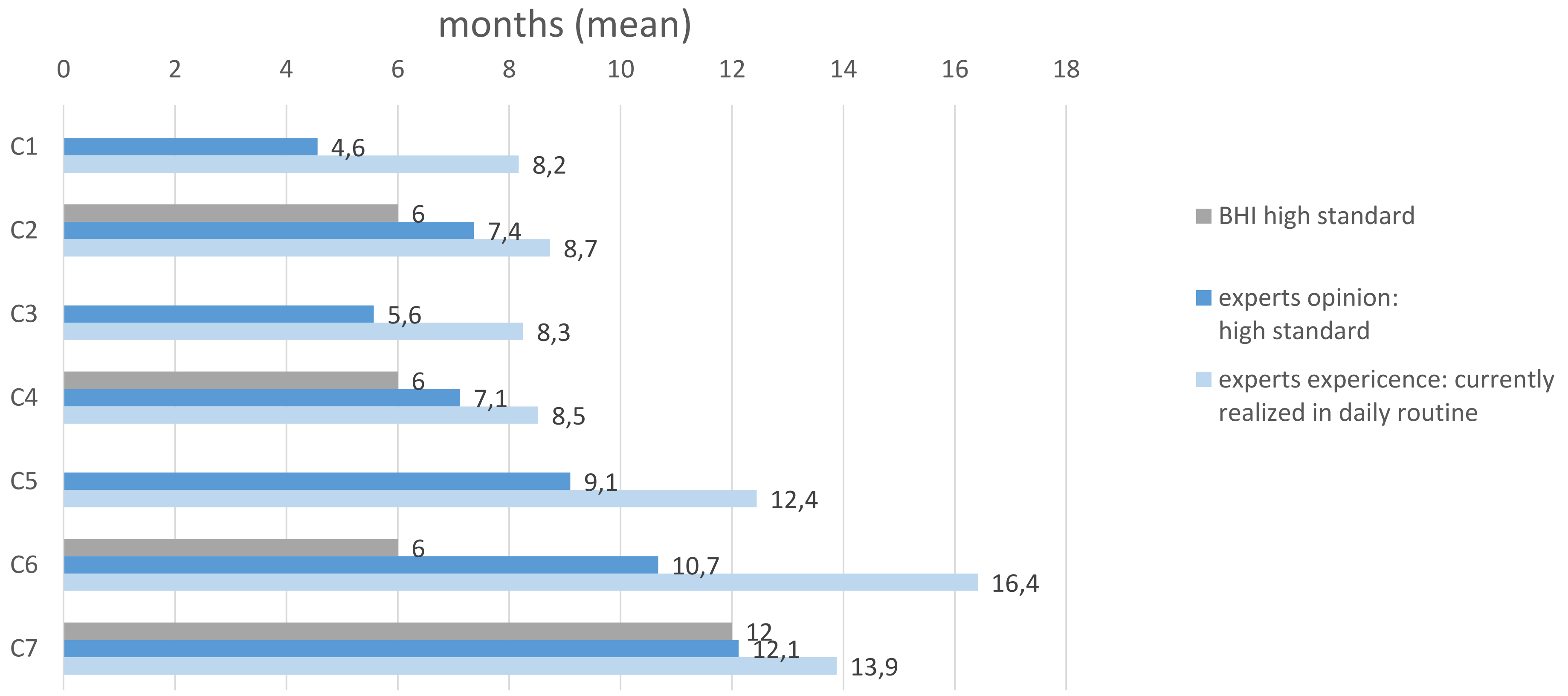

Survey: time-based quality standards of BHI

results

C. routine monitoring and support (in sum)

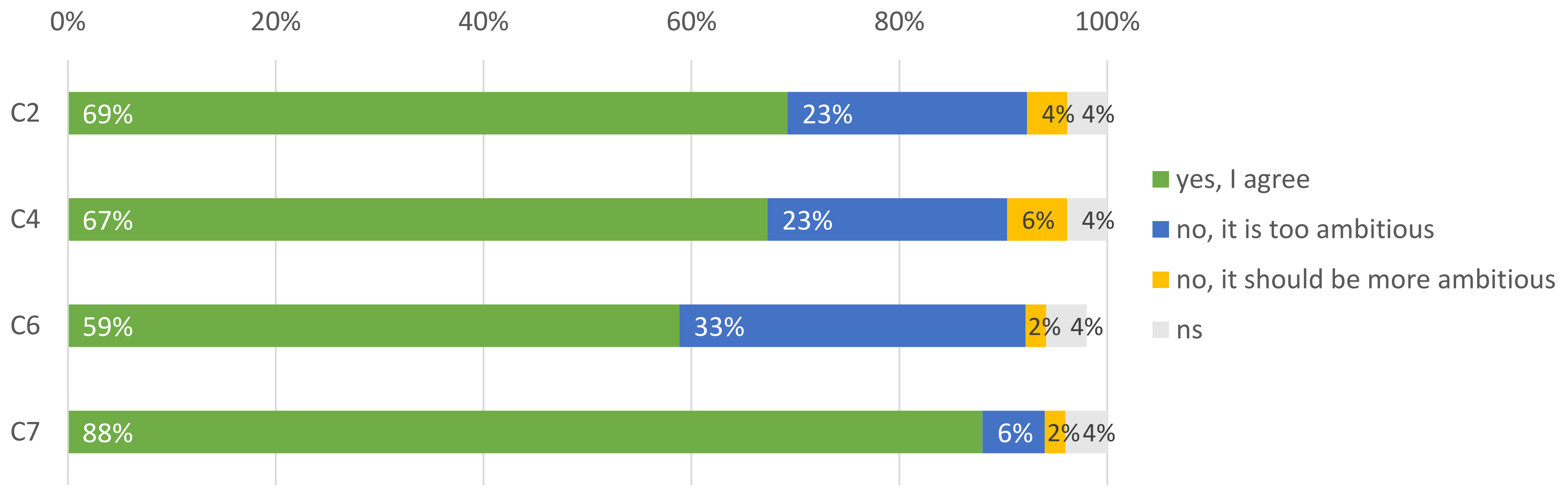

Survey: time-based quality standards of BHI

results

C. routine monitoring and support (1/8)

C1. The MS team should perform a follow-up clinical evaluation of each patient at least once every […] months. (only "good" standard of care indicated: 6 months)

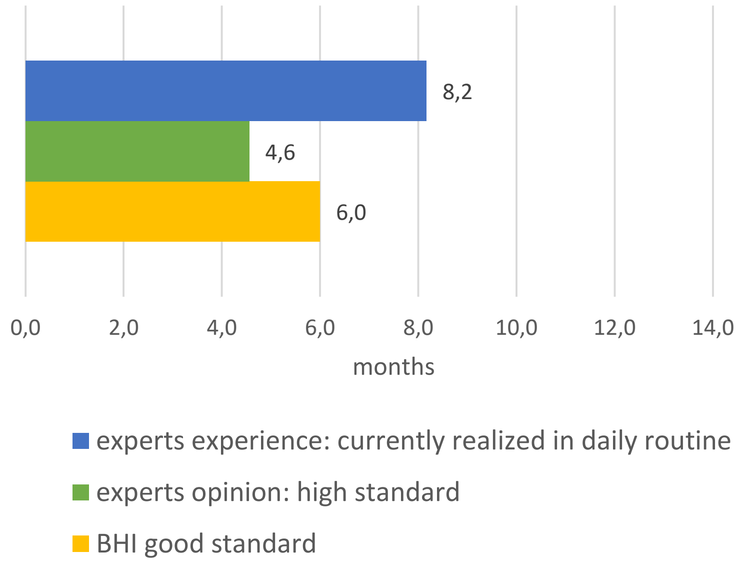

comments
Initially, contacts must be made more frequently, e.g. after a maximum of 3 months, and later every 6 months if the MS is stable.
after adjustment to medication more frequent checks - 1st month, 3rd month, 6th month
Every 3 months at the start of therapy, every 6 months over the course of therapy
A one-month follow-up can be realized in the initial phase, or in the course of high activity.
The interval depends on the activity of the disease and the tolerability of the DMTs. I have seen some patients every 3 months, but some only once a year, which can change quickly if the MS situation changes. The same applies to MRI follow-up diagnostics.
Depending on disease activity, clinical examination every 3-6 months

Survey: time-based quality standards of BHI

results

C. routine monitoring and support (2/8)

C2. The MS team should review with each patient at least once every […] months the aims of their treatment. (n=52)

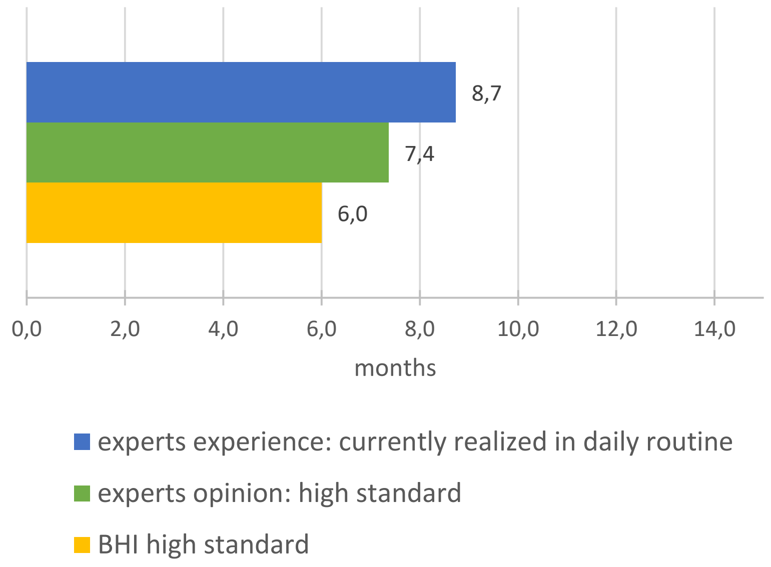

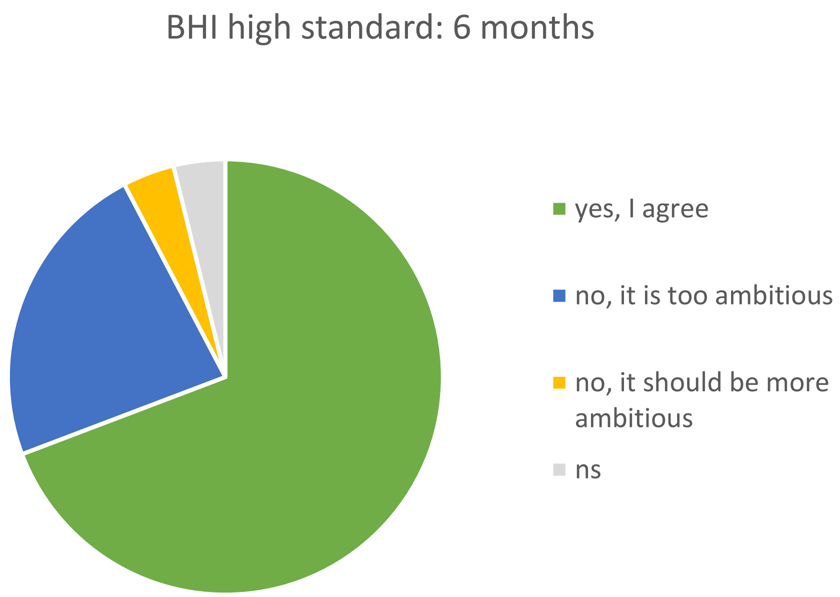

Survey: time-based quality standards of BHI

results

C. routine monitoring and support (2/8)

C2. The MS team should review with each patient at least once every 6 months the aims of their treatment. (n=52)

comments „yes, I agree“
The frequency of contact must be made dependent on the course of the MS in order to be able to care for as many patients as possible.

comments „no, it is too ambitious“
Depending on the disease activity
Or, in the case of relapse activity, everything is reconsidered regardless of the timing
The goals of MS treatment do not usually change that often. A discussion of goals should therefore take place about once a year - especially if the course is relatively stable - but also much earlier in the case of severe relapses with persistent symptoms.

Survey: time-based quality standards of BHI

results

C. routine monitoring and support (3/8)

C3. The MS team should review at least once every […] months whether each patient with MS who is not receiving a DMT is eligible for one, based on applicable guidelines. (n=50) (only "good" standard: 6 months)

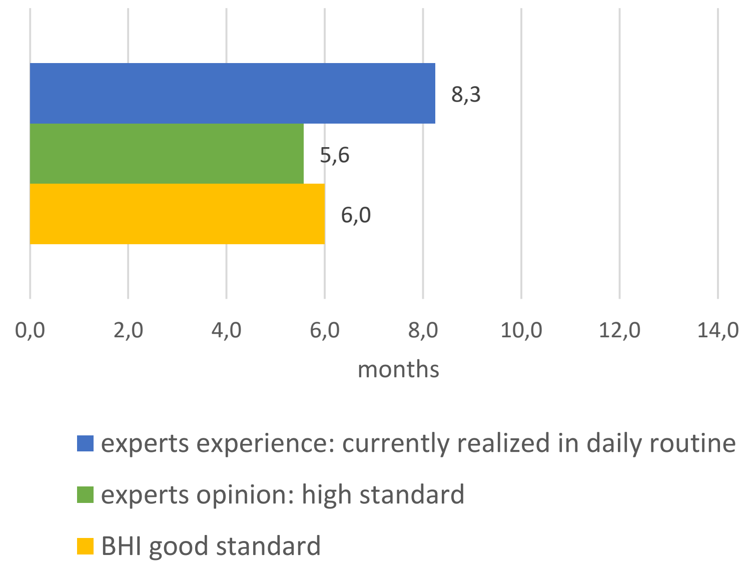

comments
There is no funding for the additional time required to fulfill this task.
This also depends on the clinical and MRI progression (cranial and spinal); regular MRIs (depending on the VB) must be performed.
This can be reconsidered at each visit, especially if new symptoms occur or after the findings of the MRI follow-up diagnostics.

Survey: time-based quality standards of BHI

results

C. routine monitoring and support (4/8)

C4. The MS team should review with each patient at least once every [...] months their currently prescribed DMT and consider alternatives if possible. (n=52)

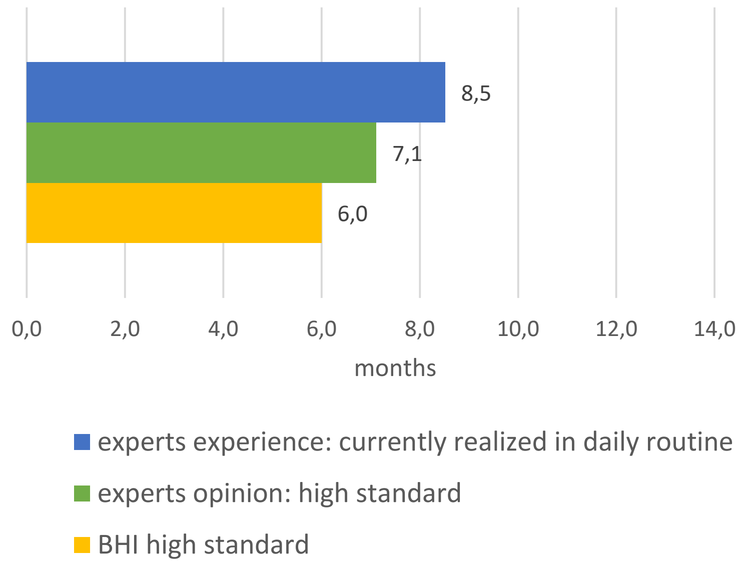

BHI high standard: 6 months

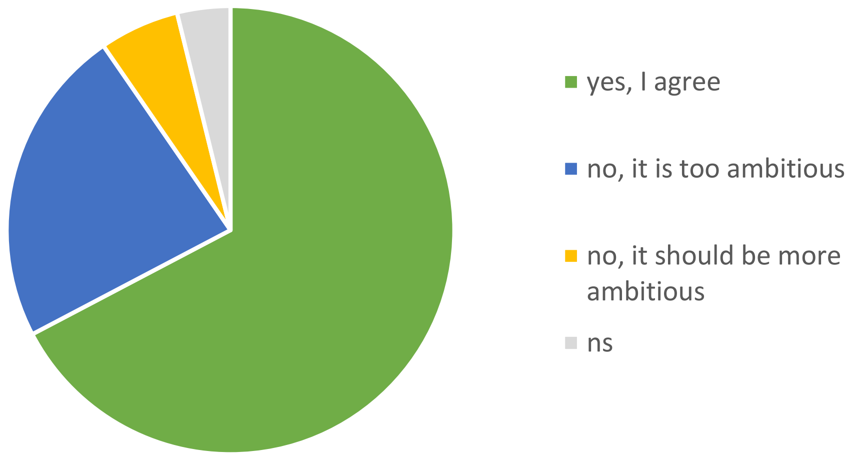

Survey: time-based quality standards of BHI

results

C. routine monitoring and support (4/8)

C4. The MS team should review with each patient at least once every 6 months their currently prescribed DMT and consider alternatives if possible. (n=52)

comments „yes, I agree“
at least in patients with an unstable course.

comments „no, it is too ambitious“
According to KV law, the doctor must be consulted and assessed once a quarter during ongoing therapy
This depends on the course of the disease and the monitoring results (clinic, MRI, laboratory)
would also depend on the course/activity of the disease!

Survey: time-based quality standards of BHI

results

C. routine monitoring and support (5/8)

C5. The MS team should engage patients with MS in an active, documented discussion about living a brain-healthy lifestyle at least once every […] months. (only "good" standard: 6 months) (n=51)

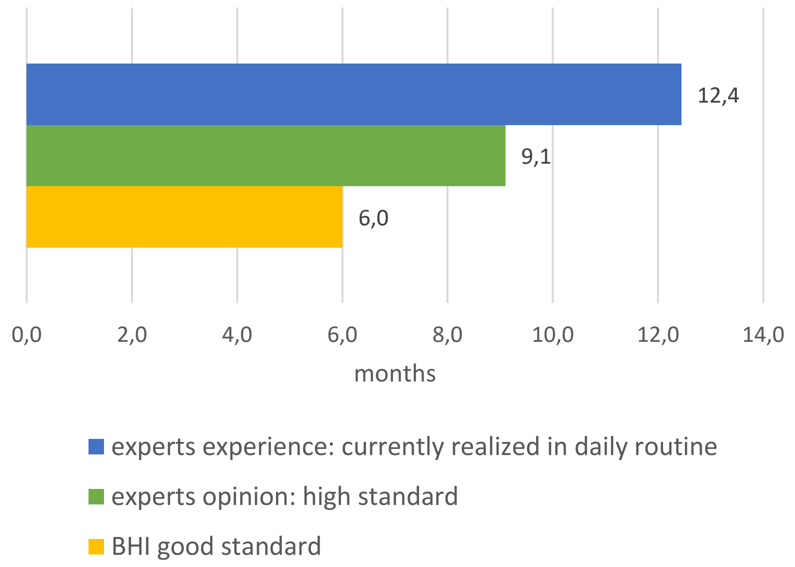

comments
Not at all, so the question of time cannot be answered.
Das kann zu jeder Visite alle 3 Monate erfolgen
ist m.E. unabhängig von festen Zeiträumen. Ich frage einen Raucher jedes Mal nach seinem Abusus und biete ihm Hilfe an; gleiches gilt für andere RF. DAs ist lediglich abhängig von der Zahl der Besuche in der Praxis.
Excessive "admonishment" tends to lead to resistance

Survey: time-based quality standards of BHI

results

C. routine monitoring and support (6/8)

C6. All patients with MS should have a check-up with an appropriate healthcare professional to screen for and/or manage comorbidities at least every […] months. (n=51)

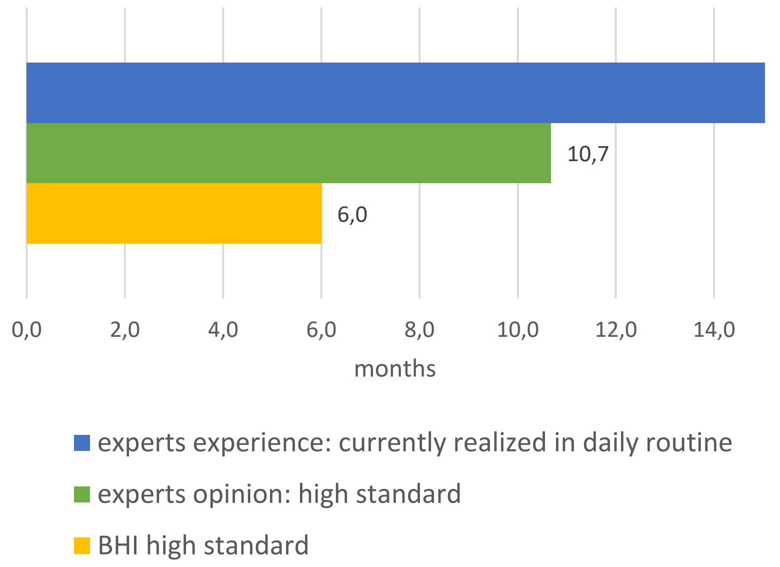

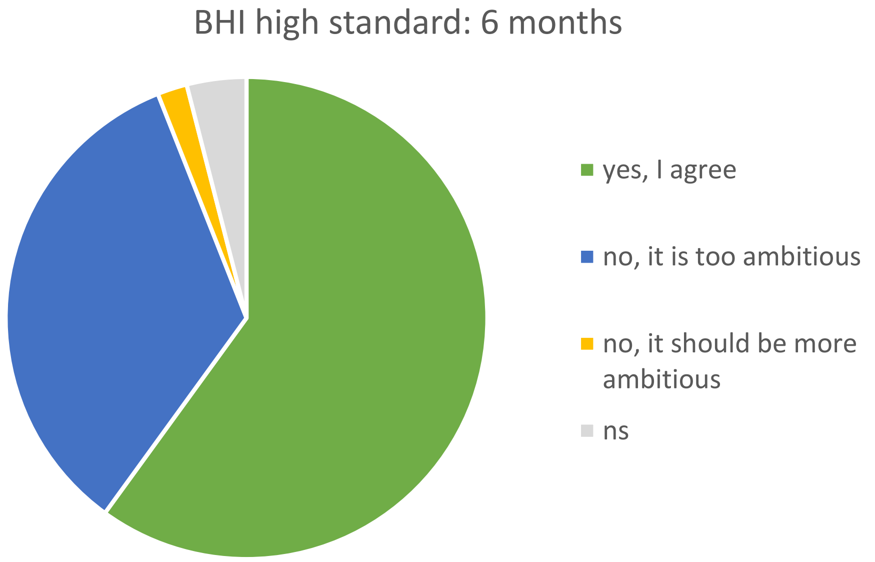

Survey: time-based quality standards of BHI

results

C. routine monitoring and support (6/8)

C6. All patients with MS should have a check-up with an appropriate healthcare professional to screen for and/or manage comorbidities at least every 6 months. (n=51)

comments „yes, I agree“
Not feasible in everyday life

comments „no, it is too ambitious“
Six-monthly check-ups with other specialists mean even more stressful appointments for patients
It depends on the age and the comorbidities
too ambitious. Which comorbidities are meant exactly? Autoimmune diseases? Vascular? ....

Survey: time-based quality standards of BHI

results

C. routine monitoring and support (7/8)

C7. All patients with MS should be offered an MRI scan at least once every […]. (n=50)

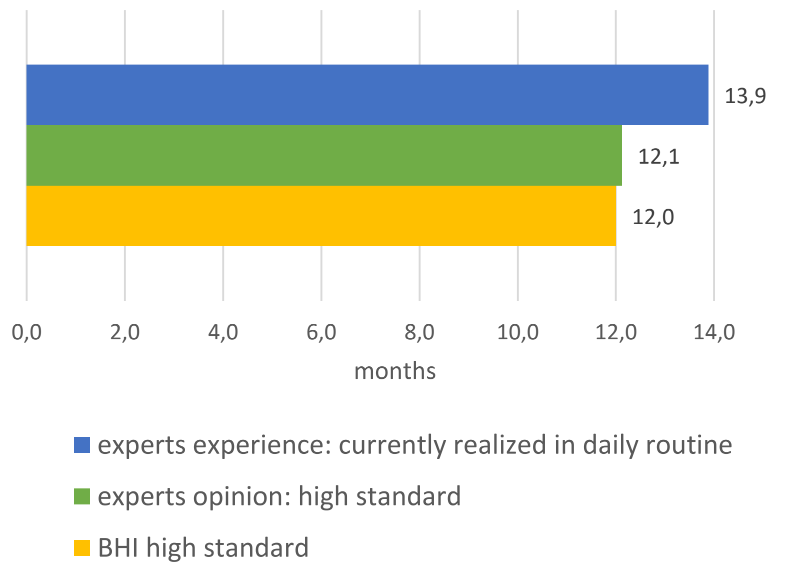

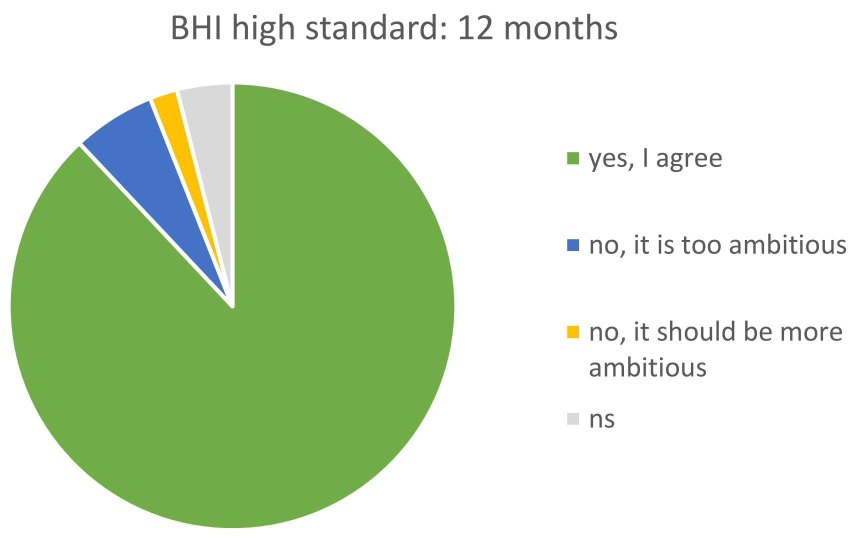

Survey: time-based quality standards of BHI

results

C. routine monitoring and support (7/8)

C7. All patients with MS should be offered an MRI scan at least once every 1 year. (n=50)

comments „yes, I agree“
the MRI frequency depends on the therapies (e.g. Natalizumab 6 months)
but also pends on therapy
For ED with a highly active course, a narrower time window should be chosen within the first few years of therapy (re-baseline, then every 6 months)
For patients who have been stable for many years, the time can also be extended
yes, but it also depends on the current and previous course of the MS.

Survey: time-based quality standards of BHI

results

C. routine monitoring and support (8/8)

Do you carry out regular MRI checks or do you have regular MRI checks carried out? (n=48)

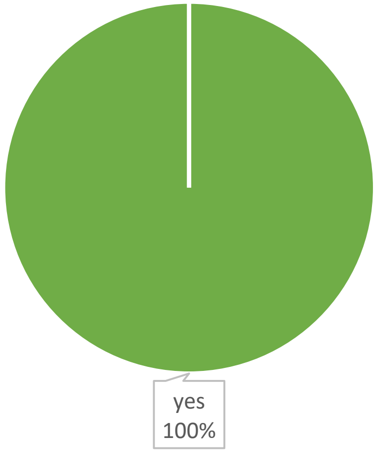

comments „yes“
the MRI frequency depends on the therapies (e.g. Natalizumab 6 months)
yes, usually once a year, but also more frequently at the start of or when switching to DMT
But no inevitability
We have it done because we are a rehabilitation clinic.

Survey: time-based quality standards of BHI

results

D. treatment decisions (in sum)

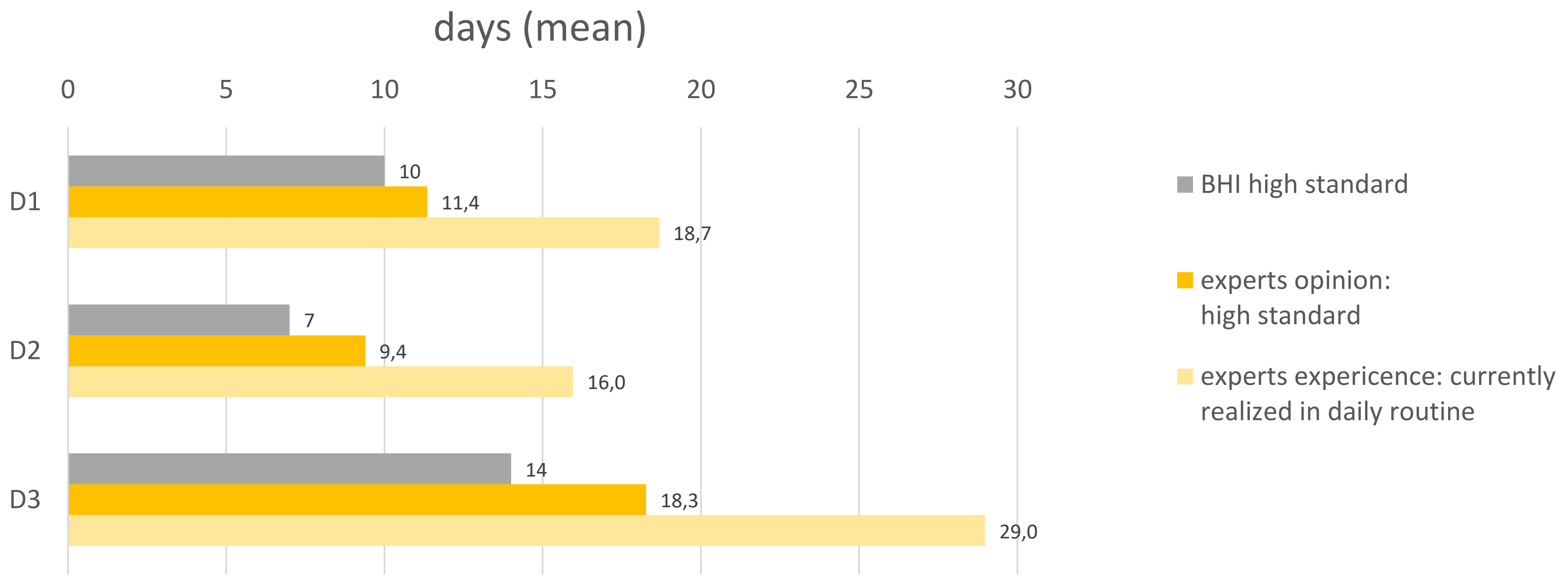

Survey: time-based quality standards of BHI

results

D. treatment decisions (in sum)

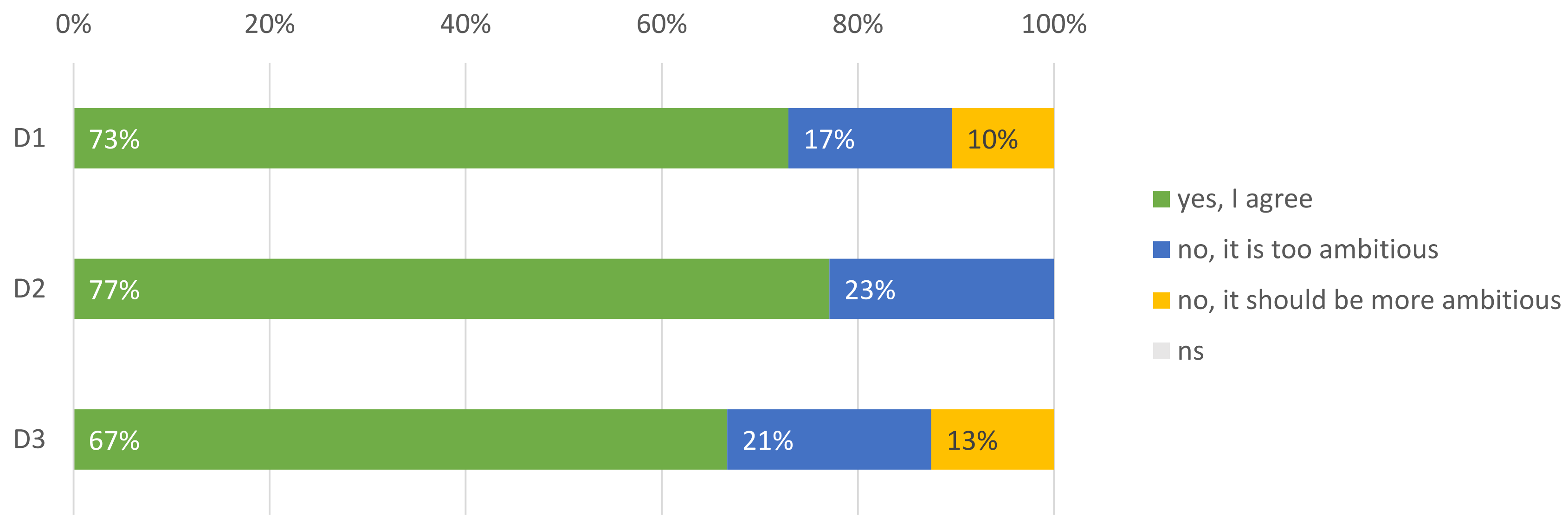

Survey: time-based quality standards of BHI

results

D. treatment decisions (1/3)

D1. Patient becomes eligible for DMT: A DMT should be offered to a patient with MS within [...] days of their becoming eligible for one. (n=48)

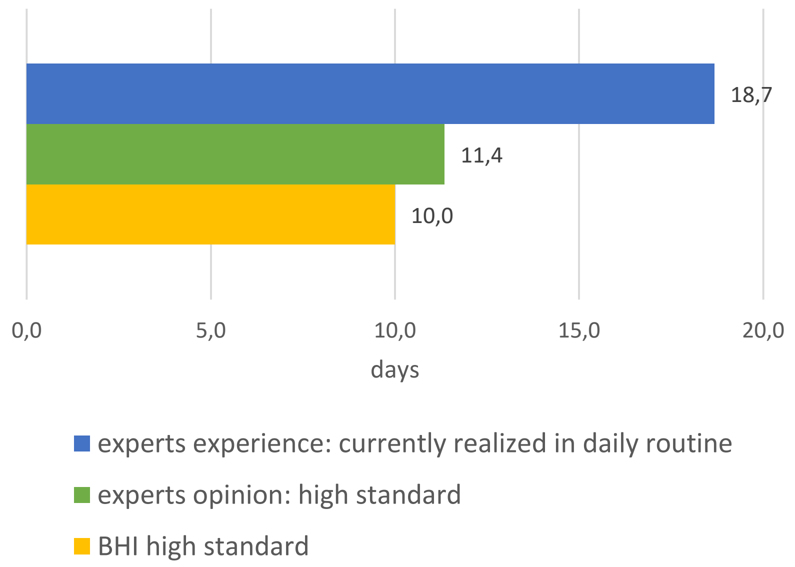

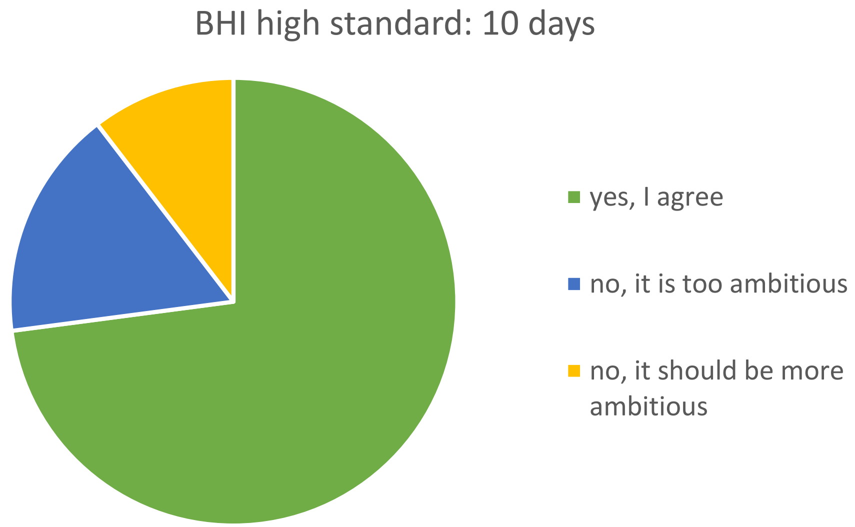

Survey: time-based quality standards of BHI

results

D. treatment decisions (1/3)

D1. Patient becomes eligible for DMT: A DMT should be offered to a patient with MS within 10 days of their becoming eligible for one. (n=48)

comments „yes, I agree“
stimme prinzipiell zu, die DMT anzubieten. Der Beginn wird dann aber wahrscheinlich noch Tage bis wenige Wochen später liegen (Vordiagnostik).

comments „no, it is too ambitious“
Within 14 days

comments „no, it should be more ambitious“
depending on clin. course, with highly active forms even more ambitious

Survey: time-based quality standards of BHI

results

D. treatment decisions (2/3)

D2. Patient decides to start DMT: Treatment with a DMT should commence within [...] days of a patient with MS agreeing this approach with their neurologist. (n=48)

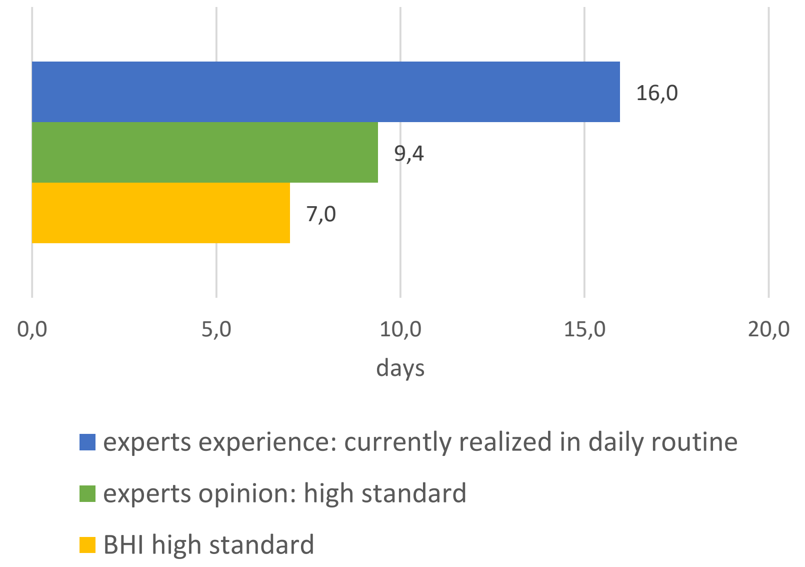

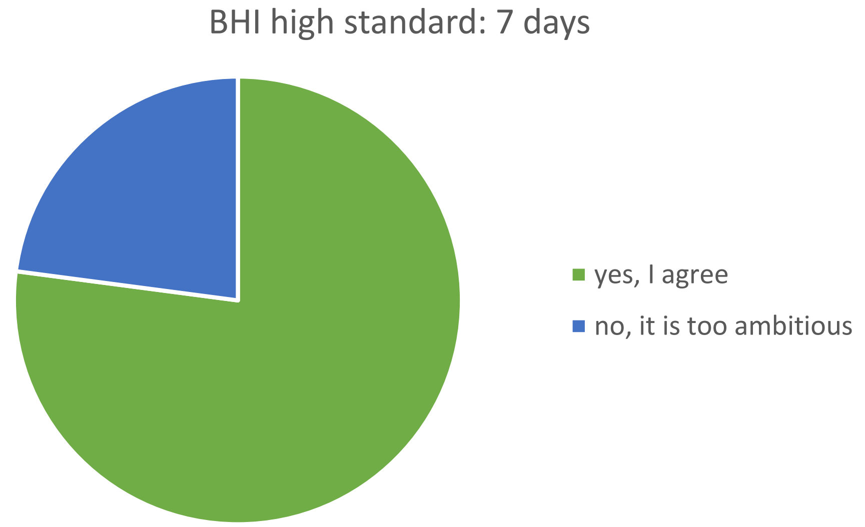

Survey: time-based quality standards of BHI

results

D. treatment decisions (2/3)

D2. Patient decides to start DMT: Treatment with a DMT should commence within 7 days of a patient with MS agreeing this approach with their neurologist. (n=48)

**comments „no, it is too ambitious“**
Preliminary examinations, vaccinations etc. necessary beforehand !
Within 14 days, especially for the highly complex infusion therapies
depending on vaccination status, missing preliminary examinations too ambitious
This is not too ambitious, but it also depends on the patient and preliminary examinations etc. In the case of a chronic illness, the 7-day period is possible in my opinion (if the patient agrees), but not necessary.
This is not always possible if appointments for infusions have to be made, sometimes 14 days can pass for logistical reasons

Survey: time-based quality standards of BHI

results

D. treatment decisions (3/3)

D3. Patient decides to start DMT: If patient's response to their current DMT is judged to be suboptimal, an appropriate alternative DMT should be offered within […] weeks. (n=48)

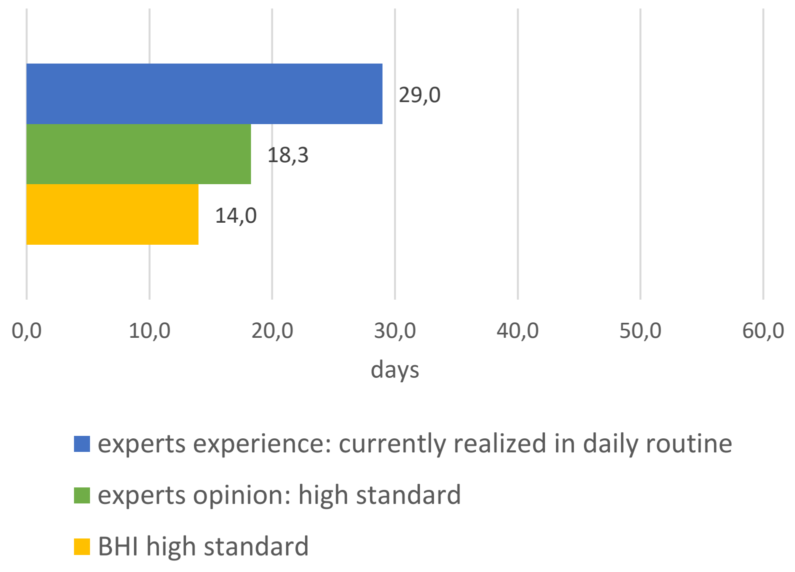

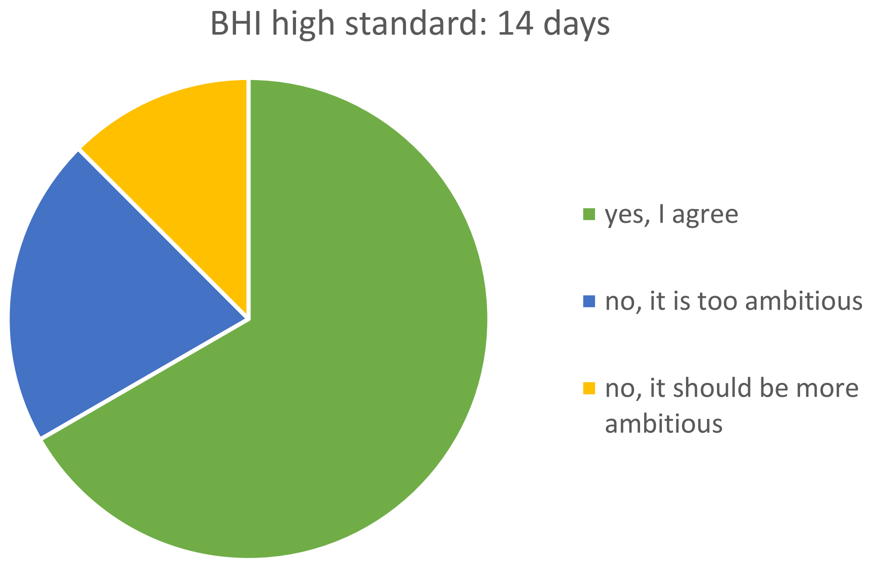

Survey: time-based quality standards of BHI

results

D. treatment decisions (3/3)

D3. Patient decides to start DMT: If patient's response to their current DMT is judged to be suboptimal, an appropriate alternative DMT should be offered within 2 weeks. (n=48)

comments „no, it is too ambitious“
the effect of the medication is rarely achieved within 14 days?
Depending on vaccination status and other preconditions
In my opinion, no neurologist can make this assessment after 14 days. Response means - in addition to tolerance - the absence of relapses or new MRI foci compared to before. As a rule, this can take place after 3 months at the earliest.
Offer yes, but changeover often takes longer because laboratory tests and treatment breaks may be necessary

Survey: time-based quality standards of BHI

results

D. new symptoms (in sum)

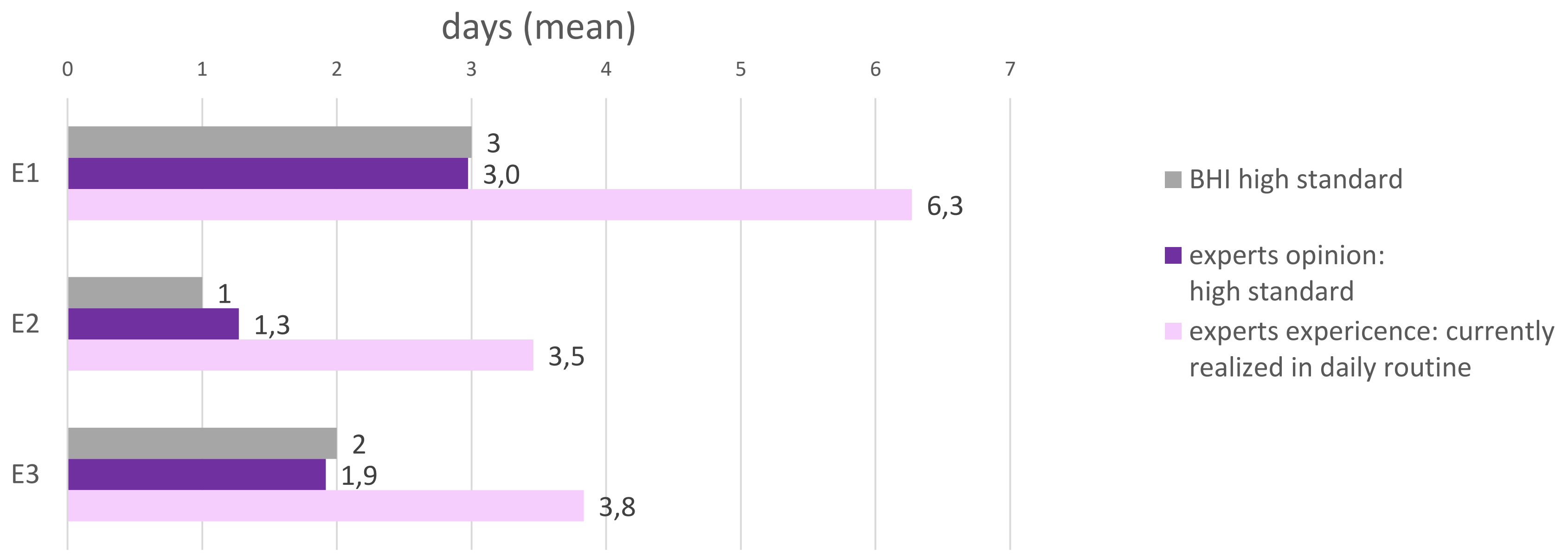

Survey: time-based quality standards of BHI

results

D. new symptoms (in sum)

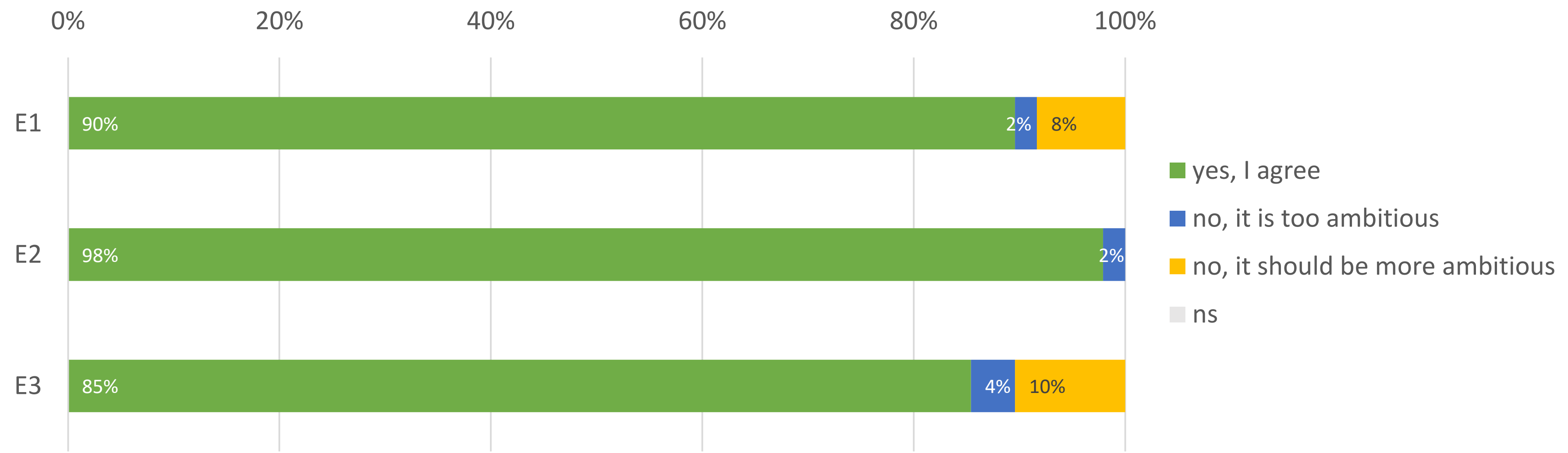

Survey: time-based quality standards of BHI

results

D. new symptoms (1/3)

E1. Patients with MS should report new or worsened symptoms to their MS team within [...] days of experiencing these symptoms. (n=48)

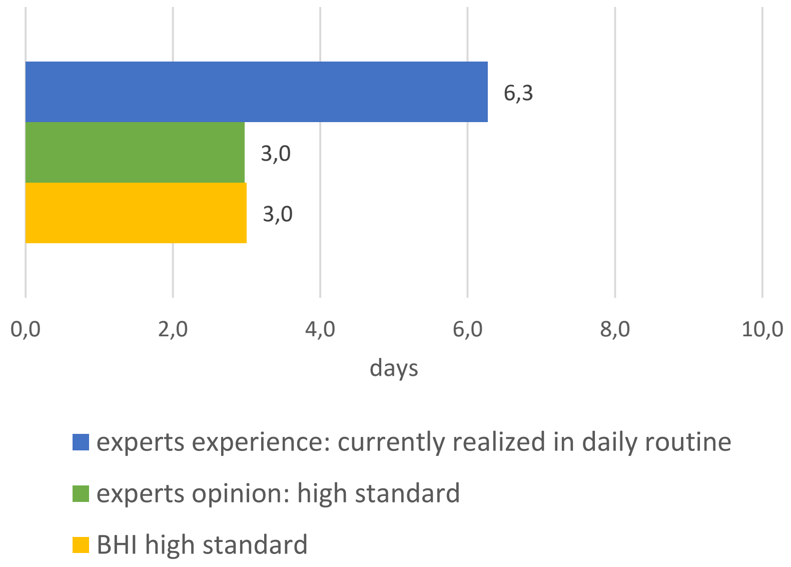

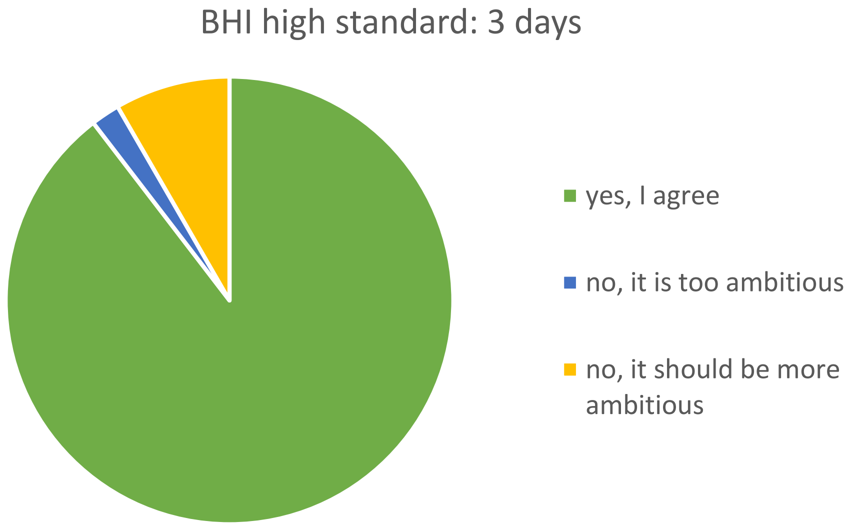

Survey: time-based quality standards of BHI

results

D. new symptoms (1/3)

E1. Patients with MS should report new or worsened symptoms to their MS team within 3 days of experiencing these symptoms. (n=48)

comments „yes, I agree“
depending on the patient
The neurologist must point this out again at every visit.

Survey: time-based quality standards of BHI

results

D. new symptoms (2/3)

E2. The MS team should respond within […] day to a patient with MS reporting an acute deterioration of symptoms. (n=48)

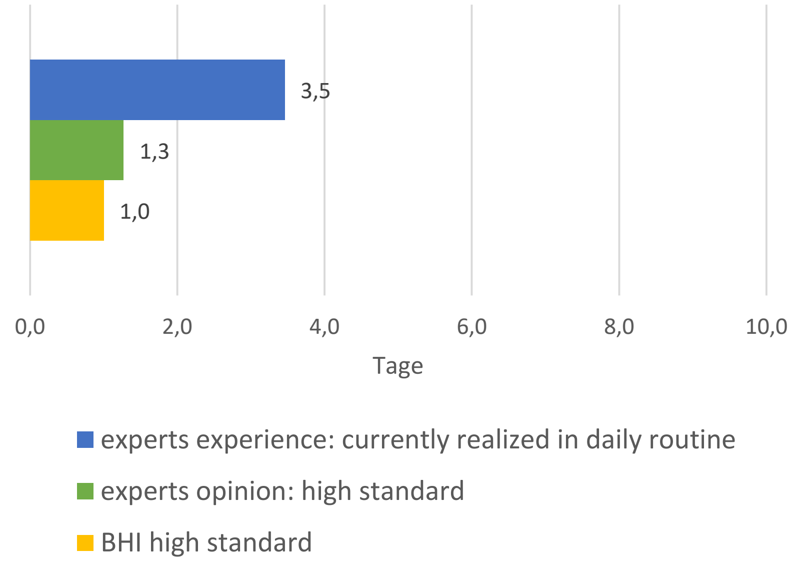

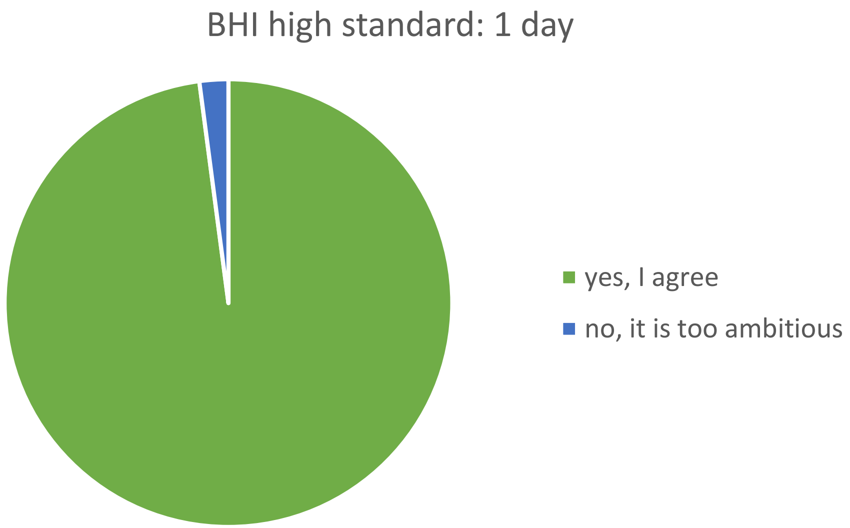

Survey: time-based quality standards of BHI

results

D. new symptoms (2/3)

E2. The MS team should respond within 1 day to a patient with MS reporting an acute deterioration of symptoms. (n=48)

comments „yes, I agree“
The neurologist should ideally see the patient on the same day and decide on additional therapies.

Survey: time-based quality standards of BHI

results

D. new symptoms (3/3)

E3. Patients with MS who experience an acute deterioration of symptoms should be seen by the relevant member of their MS team within […] days of reporting these symptoms. (n=48)

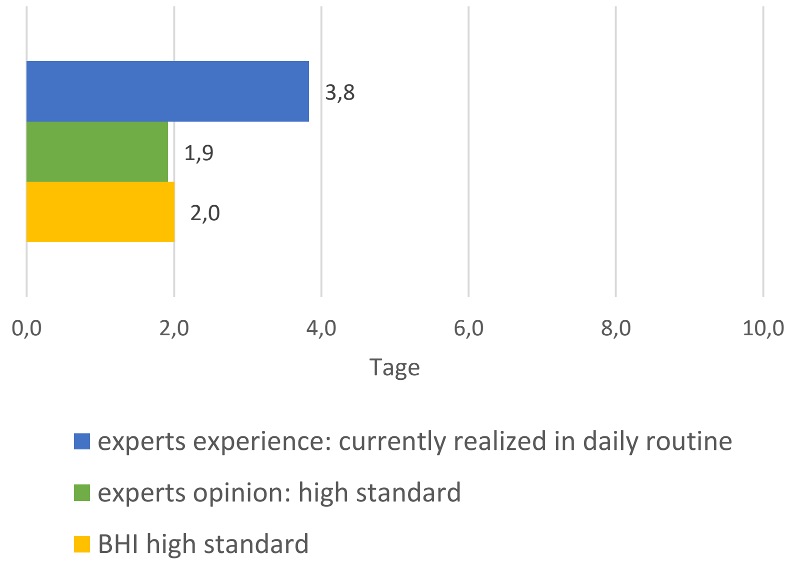

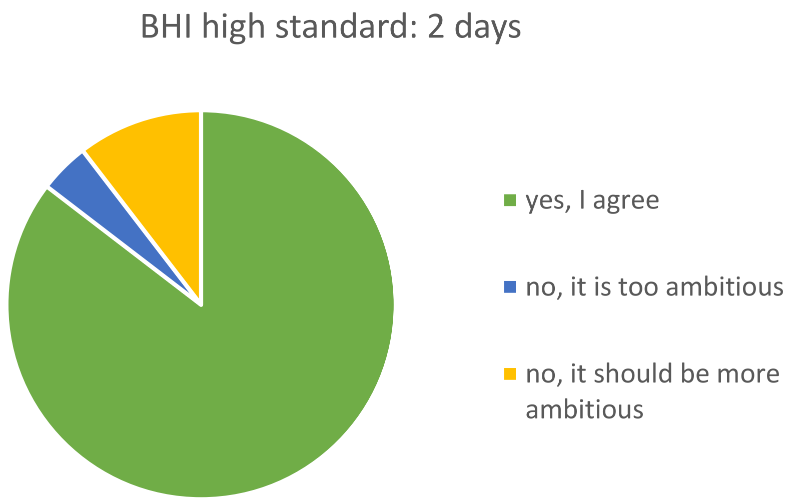

Supplement: Supplementary file 2 — Supplementary Material 2. [file 42466_2024_333_MOESM2_ESM.pdf]
